# Transcriptome analysis reveals fluid shear stress (FSS) and atherosclerosis pathway as a candidate molecular mechanism of short-term low salinity stress tolerance in abalone

**DOI:** 10.1186/s12864-022-08611-8

**Published:** 2022-05-23

**Authors:** Grace Afumwaa Boamah, Zekun Huang, Yawei Shen, Yisha Lu, Zhixuan Wang, Ying Su, Changan Xu, Xuan Luo, Caihuan Ke, Weiwei You

**Affiliations:** 1 State Key Laboratory of Marine Environmental Science, Xiamen University, Xiamen, 361102 People’s Republic of China; 2 Fujian Key Laboratory of Genetics and Breeding of Marine Organisms, Xiamen University, Xiamen, 361102 People’s Republic of China; 3 College of Ocean and Earth Sciences, Xiamen University, Xiamen, 361102 People’s Republic of China; 4 Third Institute of Oceanography, MNR, Xiamen, 361005 China; 5 College of the Environment and Ecology, Xiamen University, 361102 Xiamen, PR China

**Keywords:** Abalone, Low salinity, Interspecific hybrid, Transcriptomics, FSS pathway, Immunity

## Abstract

**Background:**

Transcriptome sequencing is an effective tool to reveal the essential genes and pathways underlying countless biotic and abiotic stress adaptation mechanisms. Although severely challenged by diverse environmental conditions, the Pacific abalone *Haliotis discus hannai* remains a high-value aquaculture mollusk and a Chinese predominantly cultured abalone species. Salinity is one of such environmental factors whose fluctuation could significantly affect the abalone’s cellular and molecular immune responses and result in high mortality and reduced growth rate during prolonged exposure. Meanwhile, hybrids have shown superiority in tolerating diverse environmental stresses over their purebred counterparts and have gained admiration in the Chinese abalone aquaculture industry.

The objective of this study was to investigate the molecular and cellular mechanisms of low salinity adaptation in abalone. Therefore, this study used transcriptome analysis of the gill tissues and flow cytometric analysis of hemolymph of *H. discus hannai* (DD) and interspecific hybrid *H. discus hannai* ♀ x *H. fulgens* ♂ (DF) during low salinity exposure. Also, the survival and growth rate of the species under various salinities were assessed.

**Results:**

The transcriptome data revealed that the differentially expressed genes (DEGs) were significantly enriched on the fluid shear stress and atherosclerosis (FSS) pathway. Meanwhile, the expression profiles of some essential genes involved in this pathway suggest that abalone significantly up-regulated calmodulin-4 (CaM-4) and heat-shock protein90 (HSP90), and significantly down-regulated tumor necrosis factor (TNF), bone morphogenetic protein-4 (BMP-4), and nuclear factor kappa B (NF-kB).

Also, the hybrid DF showed significantly higher and sustained expression of CaM and HSP90, significantly higher phagocytosis, significantly lower hemocyte mortality, and significantly higher survival at low salinity, suggesting a more active molecular and hemocyte-mediated immune response and a more efficient capacity to tolerate low salinity than DD.

**Conclusions:**

Our study argues that the abalone CaM gene might be necessary to maintain ion equilibrium while HSP90 can offset the adverse changes caused by low salinity, thereby preventing damage to gill epithelial cells (ECs).

The data reveal a potential molecular mechanism by which abalone responds to low salinity and confirms that hybridization could be a method for breeding more stress-resilient aquatic species.

**Supplementary Information:**

The online version contains supplementary material available at 10.1186/s12864-022-08611-8.

## Background

The Pacific abalone, *Haliotis discus hannai*, is a high-value aquaculture species predominantly cultured in China [1, 2]. Under aquaculture conditions, abalone encounters diverse environmental challenges such as salinity fluctuation. Particularly, abalones on farms in the coastal and inner bays could experience prolonged low salinity conditions due to freshwater intrusion [3, 4]. Moreover, abalone encounters a sudden drop in seawater salinity during severe summer rainstorms and typhoon events [5]. However, changes in ambient salinity could significantly affect abalone inflammatory and immune responses and are associated with several other physiological responses, such as disrupting electrolyte equilibrium, oxidative damage, and metabolism disorders [6–9], which eventually influence their survival and growth [10, 11].

Luckily, abalone hybrids have shown superiority in tolerating diverse environmental stresses over their purebred counterparts and have benefited the Chinese abalone aquaculture industry [10, 12–15]. However, there is a dearth of practical proof regarding the mechanisms driving their phenotypic differences to salinity tolerance.

Understanding the mechanism of low salinity tolerance is of great importance to the abalone aquaculture industry, as this would help in the future breeding of more stress-resilient abalone species. Previous studies on salinity adaptation in abalone focused on osmotic and ionic regulation, oxygen consumption, energy metabolism, and cardiac performance [5–8, 12]. Flow cytometry has also been adopted at the cellular level to assess abalone’s immune competence following salinity stress exposure [9].

Likewise, transcriptome sequencing has been adopted in the molecular study of many aquaculture organisms, including abalone [16–18], and is an effective tool to reveal the essential genes and pathways underlying the mechanism of countless biotic and abiotic stress adaptation. However, there is a dearth of evidence regarding abalone’s molecular immune defense mechanisms to salinity tolerance because previous research mainly was associated with pathogenic infections [19–21]. Besides, the few available transcriptome studies of physical stresses on abalone have focused on analyzing selected immune response candidate genes [16, 22] rather than a particular pathway and the potential crosstalk between the signaling cascades involved.

The primary objective of this study was to investigate some candidate pathways and molecular mechanisms of low salinity adaptation in abalone. Secondly, we attempt to link the data from the molecular performance with that from the biochemical and phenotype performance to create a holistic presentation of the fate of abalone during low salinity stress. Therefore, we carried out RNA sequencing (RNA-Seq) of the gill tissues during a short-term low salinity exposure to acquire a considerable lot of the abalone transcriptome and heighten our knowledge of the pathways related to low salinity-stress tolerance in abalone. Knowing how salinity variation influences the signaling cascades, the crosstalk between the various signaling cascades, and how the abalone expresses each of the genes involved would help us understand how the pathway functions in the abalone.

For several reasons, the gill of abalone was chosen as the most appropriate organ for RNA sequencing. For instance, the gill of abalone is in direct contact with the aquatic environment and is exposed to several conditions capable of causing stress, such as pathogens, pollutants, and alterations in salinity, temperature, and oxygen levels [16, 23]. For most aquatic organisms, the gills play an essential role in breathing, filtration, and aid immunity [24]. In bivalves, the gill is the main organ for calcium uptake from the water, acting as a “calcium sink” in others [25]. Gills are also the primary sites for peripheral systemic osmosensors in fish and a vital organ for osmoregulation in crustaceans [26, 27]. Besides, much more pathways and differentially expressed genes were found in the gill than in the muscle of some aquatic invertebrates during salinity challenges [26, 28].

In addition, abalone hemolymph, which also plays a crucial role in their immune system [18, 29], was sampled to confirm some immune-related activities such as total hemocyte count (THC), phagocytosis (PHA), and reactive oxygen species production (ROS) production via flow cytometric analysis. Phenotype data on survival and growth was acquired after long-term exposure to various salinities.

This study provides new evidence of the molecular mechanism of low salinity adaptation in abalone.

## Results

No mortalities were observed during the period of acclimation and short-term exposure to low salinity.

### Short-term effects of low salinity on molecular response

#### Overview of RNA-Seq data analysis

After quality control, we obtained a total of 118.64 Gb clean reads. Moreover, Q20 exceeded 96%, and Q30 exceeded 90% in both species, indicating high-quality data for the subsequent bioinformatics analysis. These clean reads were then aligned to the *H. discus hannai* reference genome. The reads mapping ranged from 84.26%-85.60% in DD and 63.02%-65.19% in DF, respectively. Also, over 75% and over 55% of unique mapped reads were observed in DD and DF (Supplementary Table 1).

**Fig. 1:**
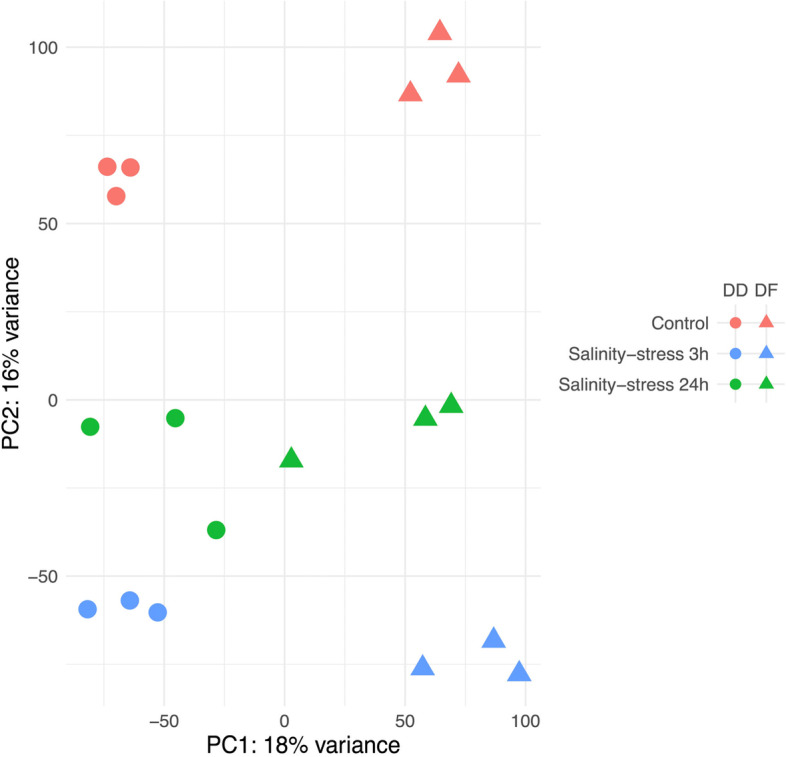
Principal Component Analysis Results Map (PCA) for gill tissues of abalone showing separation between the controls (red color), the low salinity-stressed groups at 3 h (blue color) and 24 h (green color), and between the species: *Haliotis discus hannai* (DD; circles), hybrid *H. discus hannai* ♀ × *H. fulgens* ♂ (DF, triangles)

Furthermore, a Principal Component Analysis (PCA) for all samples, based on FPKM values, suggests a separation between the control and low salinity-treatment samples along PC2 (Fig. 1). Meanwhile, after 24 h under low salinity, samples were observed to be approaching the control group along PC2, indicating some recovery. A clear separation between DD and DF was also observed along PC1. Moreover, clustering was observed within biological replicates. Also, the Pearson correlation coefficient between biological replicates, based on the Reads Per Kilobases per Million reads (FPKM) values, were larger than 0.8 in both species (Figure not shown).

**Fig. 2:**
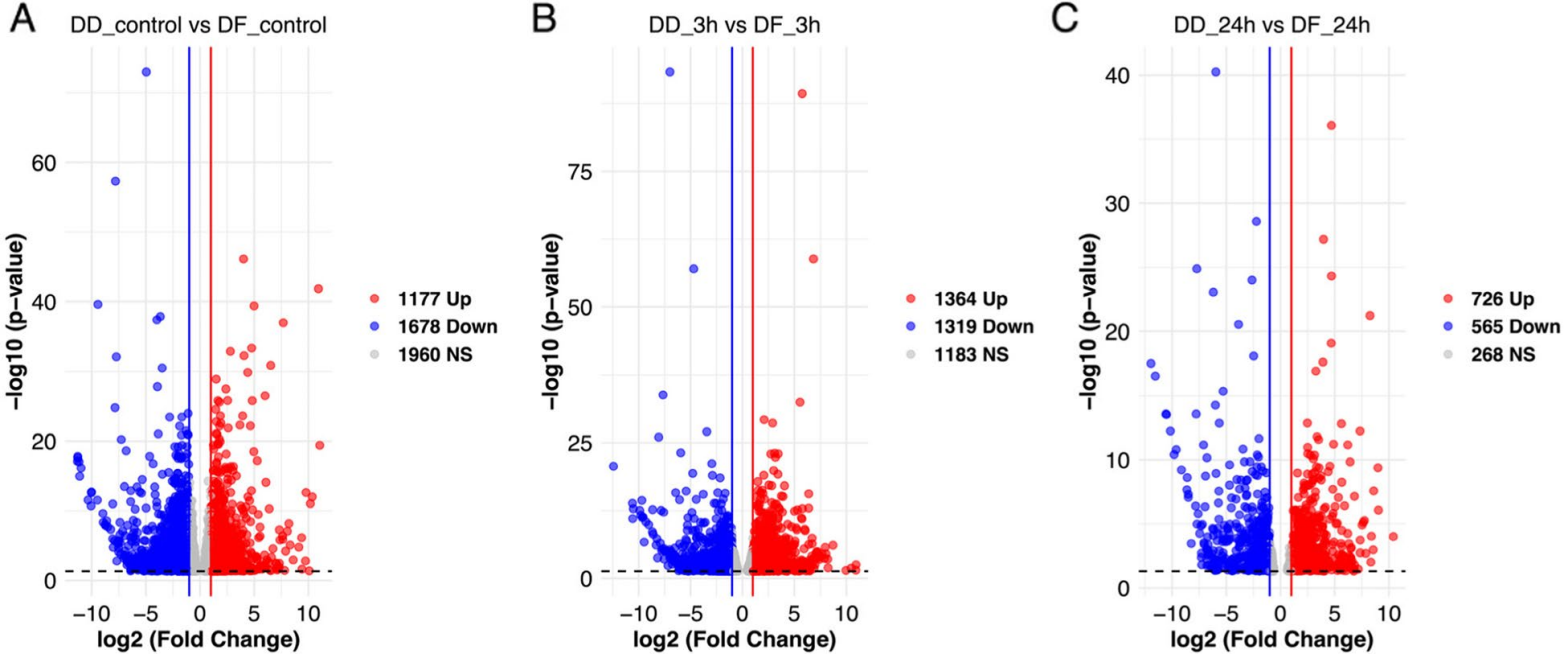
Volcano plots of the differentially expressed genes (DEGs) of gill tissues of abalone during short-term low salinity exposure: *H. discus hannai* (DD) and hybrid *H. discus hannai* ♀ × *H. fulgens* ♂ (DF). **A** Comparison between DD and DF at the control, **B** Comparison between DD and DF after 3 h at low salinity, and **C** Comparison between DD and DF after 24 h at low salinity

Identification and analysis of differentially expressed genes: Comparison of gene expression levels revealed 4930 DEGs in DD, 6651 DEGs in DF, and 8121 between the DD and DF (Supplementary Fig. 1). Relative to the control, 1012 and 1101 DEGs were up-and down-regulated after 3 h while 903 and 938 DEGs were up-and down-regulated after 24 h in DD (Supplementary Fig. 2I). In DF, 1640 and 1758 DEGs were up and down-regulated after 3 h while 1009 and 1187 DEGs were up-and down-regulated in DF after 24 h (Supplementary Fig. 2II). Also, a comparison between DD and DF revealed 1177 and 1678 up-and down-regulated DEGs at control, 1364 and 1319 up-and down-regulated DEGs after 3 h, and 726 and 565 up-and down-regulated DEGs after 24 h (Fig. 2).

**Fig. 3:**
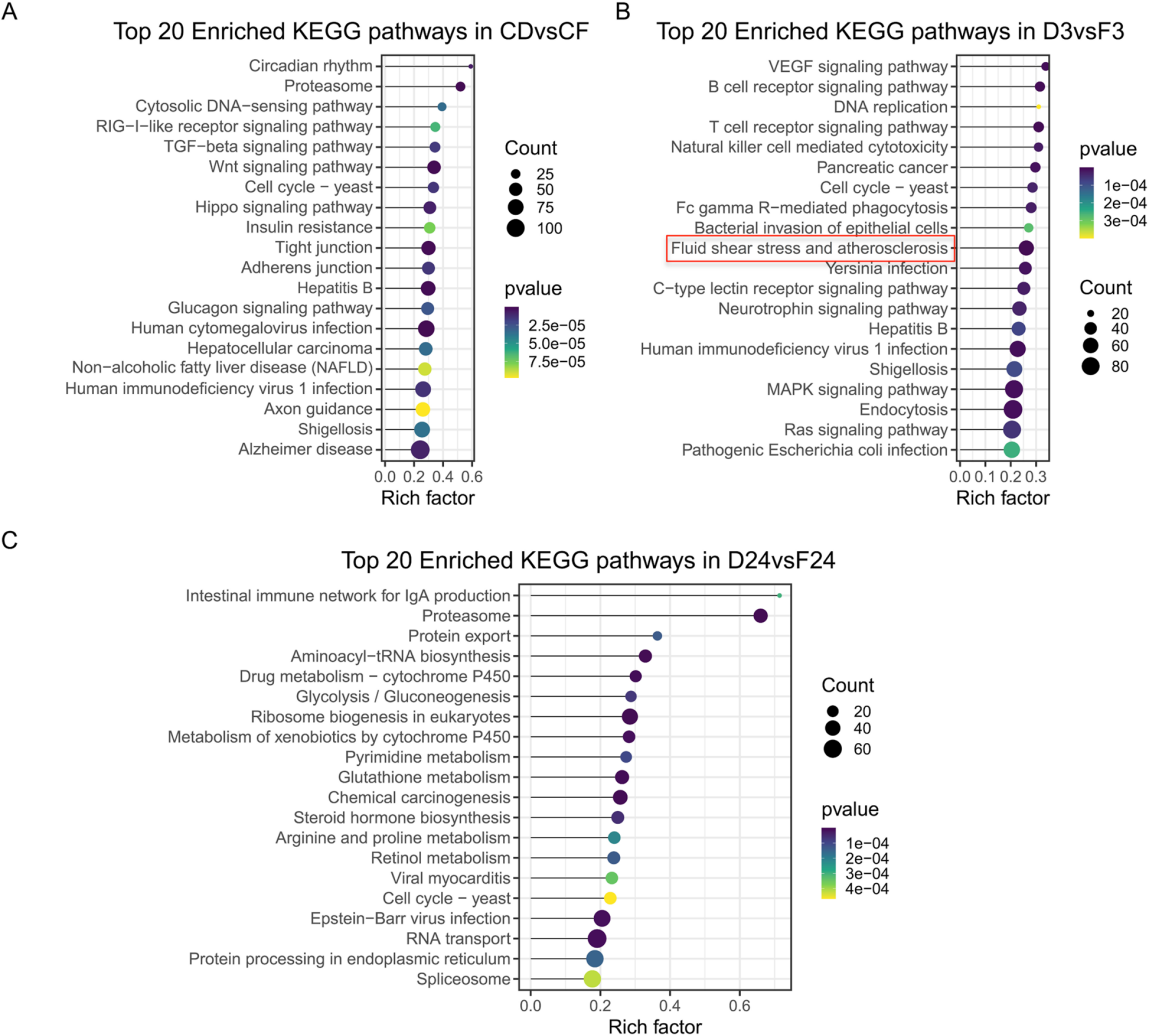
Comparison of top 20 Kyoto Encyclopedia of Gene and Genome (KEGG) pathways enrichment statistics of gill tissues of abalone during short-term low salinity exposure. The FSS pathway is highlighted with a rectangular red box. *H. discus hannai* (DD) and hybrid *H. discus hannai* ♀ × *H. fulgens* ♂ (DF). **A** Comparison between control DD (CD) and control DF (CF), **B** Comparison between low salinity groups of DD after 3 h (D3) and DF after 3 h (F3), and **C** Comparison between low salinity groups of DD after 24 h (D24) and DF after 24 h (F24). The size of each point represents the number of genes annotated to the KEGG pathway. Different colors from yellow to mauve represent the *p*-value of the enrichment

KEGG pathway enrichment results: The DEGs in DD and DF, under low salinity exposure, were involved in several pathways, including fluid shear stress (FSS) and atherosclerosis, apoptosis, necroptosis, and Foxo signaling pathway, as revealed by the KEGG pathway enrichment analysis (Supplementary Fig. 3). Furthermore, a comparison between DD and DF at 3 h (D3 vs F3) revealed that the FSS pathway, the pathway of interest in this study, was one of the top 20 enriched pathways (Fig. 3).

#### qRT-PCR verification of the FSS pathway genes expression

**Fig. 4:**
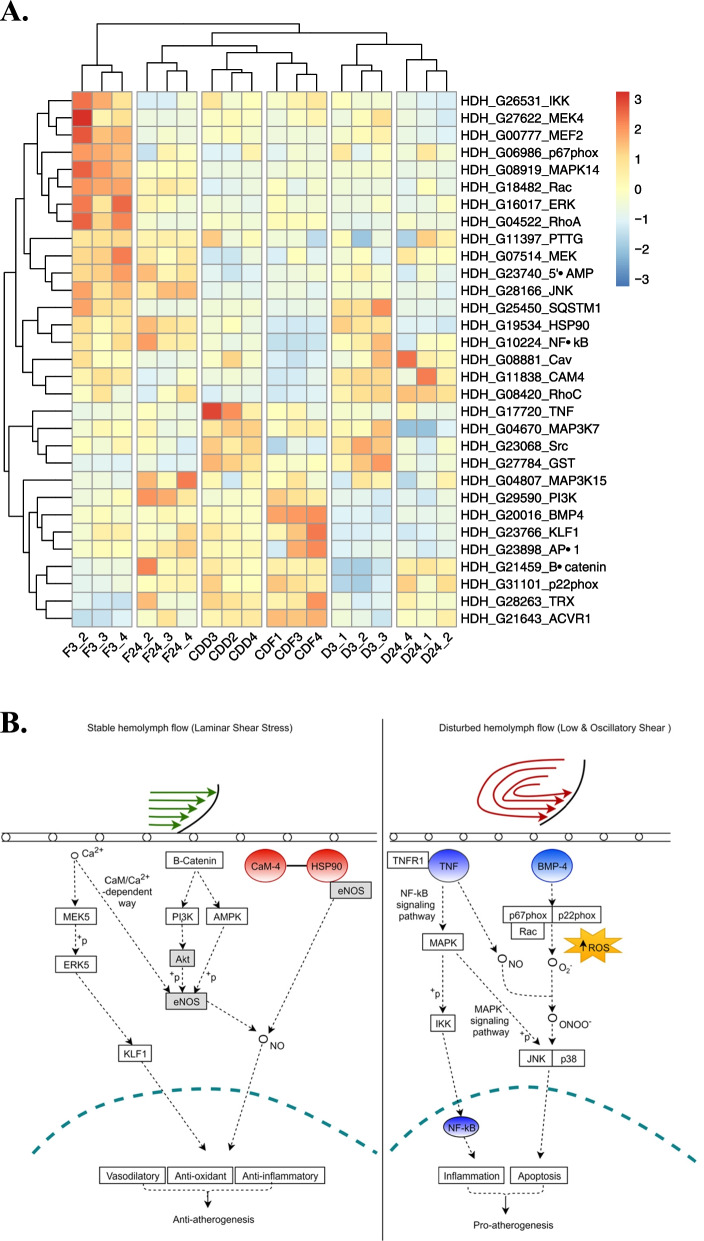
**A** Heat map showing the expression pattern of some genes involved in the FSS pathway in abalone. **B** Hypothesized model of the FSS pathway in abalone showing crosstalk between some genes (adapted from Kanehisa and Goto [30]). Hypothetically, up-regulation of all the genes under stable hemolymph flow (on the left) should lead to gill ECs anti-oxidation and anti-inflammation, while up-regulation of all the genes under disturbed hemolymph flow (on the right) should lead to gill ECs inflammation and apoptosis. Oval shapes denote genes that were validated by qRT-PCR: Red color denotes genes that were up-regulated in expression relative to the control. Blue color denotes genes that were down-regulated in expression relative to the control. Grey-colored rectangular boxes denote genes that were not expressed in the current study

**Fig. 5:**
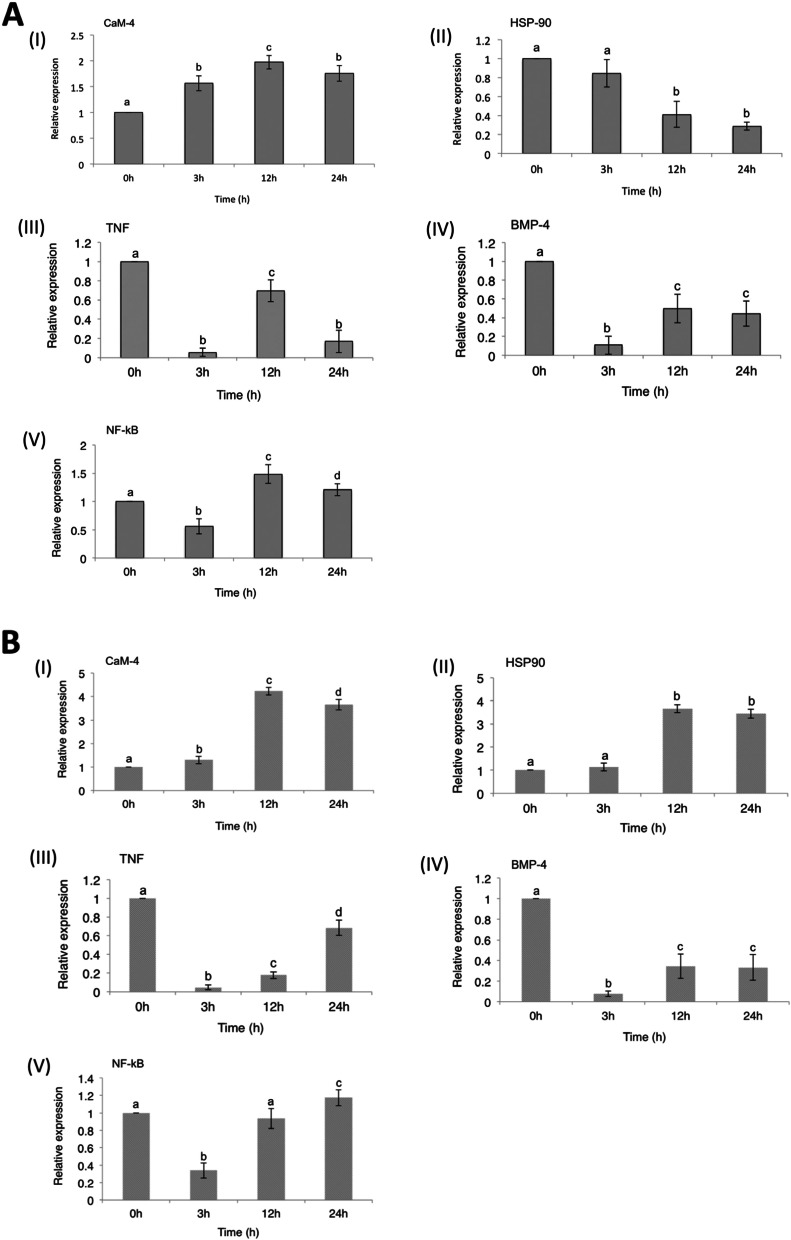
Expression profile of five FSS pathway genes in abalone gills during short-term hyposmotic stress: **A** The Pacific abalone *Haliotis discus hannai* (DD), and **B** Hybrid *H. discus hannai* ♀ × *H. fulgens* ♂ (DF), (I) Calmodulin-4, (II) Heat shock protein90, (III) Tumor necrosis factor, (IV) Bone morphogenetic protein-4, and (V) Nuclear factor kappa-B. Sampling times = “0 h”: control group; “3 h”, “12 h”, and “24 h”: exposure times at low salinity. Statistical analysis was performed by One-way ANOVA, followed by Turkey’s HSD test. Different alphabets denote a significant difference between sampling times. Differences were deemed significant at *P* < *0.05*

**Fig. 6:**
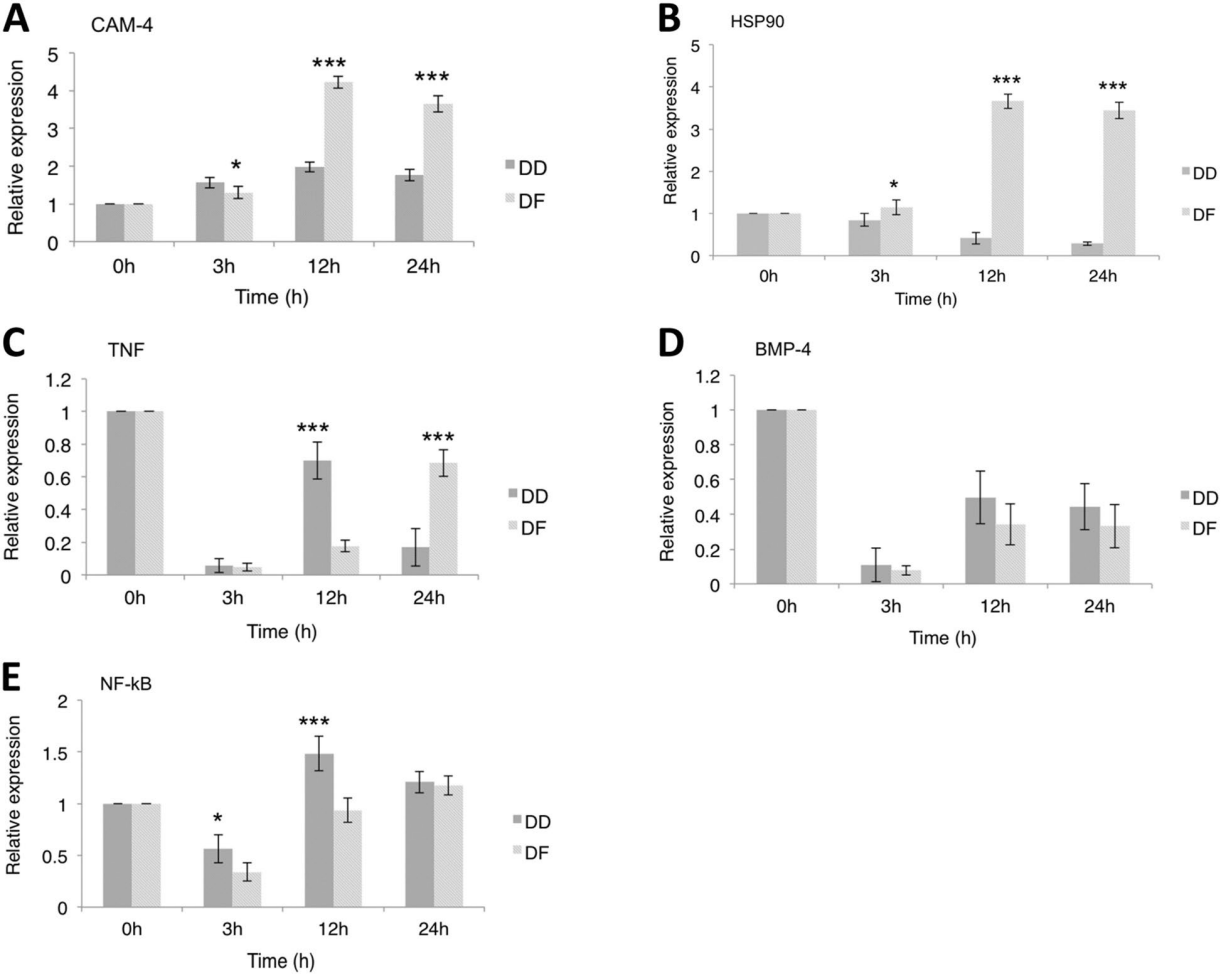
Comparison of the gene expression profiles between *Haliotis discus hannai* (DD) and hybrid *H. discus hannai* ♀ × *H. fulgens* ♂ (DF) during short-term low salinity stress: **A** Calmodulin-4, **B** Heat shock protein90, **C** Tumor necrosis factor, **D** Bone morphogenetic protein-4, and **E** Nuclear factor kappa-B. Sampling times = “0 h”: control group; “3 h”, “12 h”, and “24 h”: exposure times at low salinity. Statistical analysis was performed by Welch’s t-test at a given sampling time. Differences were deemed significant at *P* < *0.05.* “*”: *P* = *0.01*; “**”: *P* = *0.004*; “***”: *P* = *0.000*

The heat map (Fig. 4A) shows that low salinity significantly regulated all the FSS pathway genes, including the five genes validated by qRT-PCR time-dependently. A hypothesized model of the FSS pathway in abalone and the crosstalk between these five genes and other genes (adapted from Kanehisa and Goto [30] is presented in Fig. 4B. Firstly, the expression of calmodulin (CaM) remained significantly up-regulated (*P* < *0.05*) in both species throughout the low salinity exposure (Fig. 5A&B, I). Meanwhile, its expression suggests a two-phase pattern with a significant peak expression at 12 h. Also, DD recorded a significantly higher (*P* = *0.01*) expression of CaM at 3 h, while its expression was significantly higher in DF at 12 h and 24 h (*P* = *0.000*) (Fig. 6A).

The expression of heat-shock protein90 (HSP90) showed no significant difference at 3 h relative to the control (Fig. 5A&B, II). However, at 12 h and 24 h, a significant down-regulation was observed in DD while a significant up-regulation was observed in DF. Also, HSP90 was significantly up-regulated in DF than in DD at 3 h (*P* = *0.01*), 12 h, and 24 h (*P* = *0.000*) (Fig. 6B).

Contrary to the expression pattern of CaM and HSP90, tumor necrosis factor (TNF) was significantly down-regulated (*P* = *0.000*) throughout the hypo-osmotic exposure period (Fig. 5A&B, III). Meanwhile, DD observed a sharp rise in TNF expression at 12 h, which declined at 24 h. However, the expression profile in DF assumed a gradual rise with time. Between the species, TNF expression was significantly higher in DD at 12 h (*P* = *0.000*) and significantly higher in DF at 24 h (*P* = *0.000*), though all expressions were below the control levels (Fig. 6C).

The expression profile of bone morphogenetic protein (BMP) was not different from that of TNF, showing a significant (*P* = *0.000*) down-regulation throughout the hypo-osmotic exposure period (Fig. 5A&B, IV). Meanwhile, a gradual rise in its expression was observable at 12 h and 24 h, with a significant difference relative to its expression at 3 h. There was no significant difference (*P* > *0.05*) between DD and DF regarding the expression of BMP at any time point (Fig. 6D).

Finally, nuclear factor kappa B (NF-kB) was significantly (*P* = *0.000*) down-regulated at 3 h during low salinity exposure in DD (Fig. 5A, V), but was significantly up-regulated at 12 h (*P* = *0.000*) and 24 h (*P* = *0.03*). Similarly, DF recorded a significant (*P* = *0.000*) down-regulation in the expression of NF-kB at 3 h under low salinity exposure, followed by a rise to control levels at 12 h, and a significant (*P* = *0.01*) up-regulation at 24 h (Fig. 5B, V). Between the species (Fig. 6E), NF-kB expression was significantly higher in DD than in DF at 3 h (*P* = *0.01*) and 12 h (*P* = *0.000*).

### Short-term effects of low salinity on cellular immune responses

**Fig. 7:**
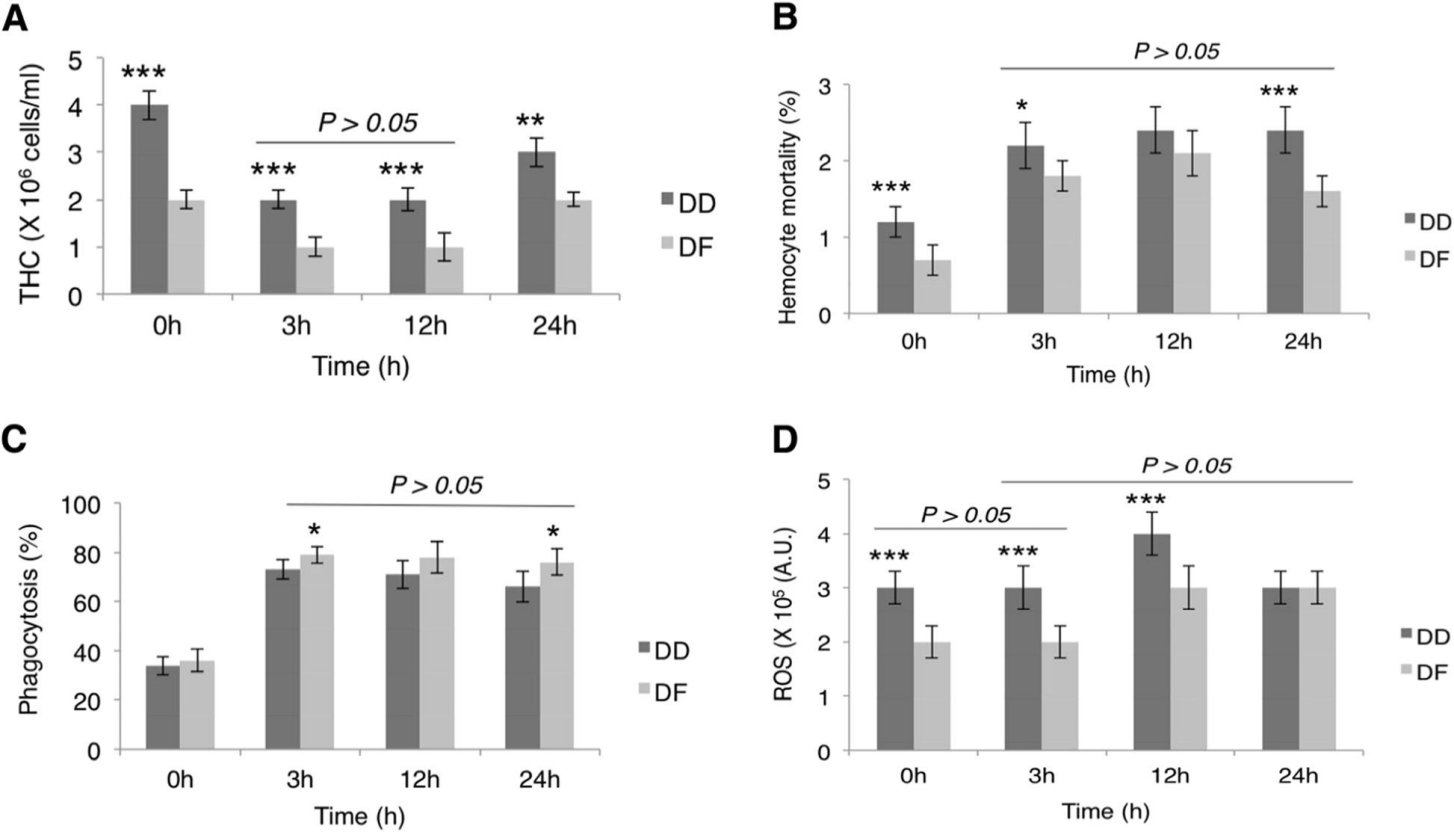
Flow cytometry analysis of *Haliotis discus hannai* (DD) and hybrid *H. discus hannai* ♀ × *H. fulgens* ♂ (DF) hemocytes during short-term hyposmotic stress: **A** Total hemocyte count (THC), **B** Hemocyte mortality, **C** Reactive oxygen species (ROS), and **D** Phagocytosis. “0 h”: control group; “3 h”, “12 h”, and “24 h”: exposure times at low salinity. One-way ANOVA, followed by Turkey’s HSD test, was used to analyze the differences between the sampling times for each species. Analysis of the differences between the species at a given sampling time was done by Welch’s t-test. Asterisks = significant difference between DD and DF at the given sampling time: “*”: *P* = *0.02*; “**”: *P* = *0.002*; “***”: *P* = *0.000.* The bar indicates no significant difference between sampling times (*P* > *0.05*) for the individual species

Total hemocyte count (THC): The Pacific abalone recorded a significantly higher (*P* < *0.05*) total hemocyte count (THC) at the control and during low salinity exposure than DF (Fig. 7A). Both species showed a marked decline (*P* = *0.000*) in THC at three hours and 12 h under low salinity exposure compared to the control. At 24 h, THC showed a trend of gradual recovery to control levels and was significantly higher than that recorded at 3 h and 12 h (*P* = *0.000*) but significantly lower than the control (*P* = *0.01*). There was no significant difference between THC at 3 h and 12 h (*P* = *0.9*).

Hemocyte mortality: Hemocyte mortality remained significantly higher than the control (*P* = *0.000*) during low salinity exposure with no significant difference between 3 h, 12 h, and 24 h (*P* > *0.05*) (Fig. 7B). Again, DD observed significantly higher basal hemocyte mortality than DF (*P* = *0.000*), as well as at 3 h (*P* = *0.02*) and 24 h (*P* = *0.000*).

Phagocytosis: As shown in Fig. 7C, % phagocytosis significantly increased under low salinity exposure (*P* = *0.000*) relative to the basal levels. Meanwhile, % phagocytosis did not significantly differ between the two species at the basal level but was significantly higher in DF at 3 h and 24 h (*P* = *0.02*). No significant difference was observed between 3 h, 12 h, and 24 h (*P* > *0.05*).

Reactive oxygen species (ROS): Lastly, the production of reactive oxygen species (ROS) showed no marked difference from the control at 3 h (*P* > *0.05*). However, ROS production displayed an increase at 12 h and 24 h, and there was no significant difference between 3 h, 12 h, and 24 h. DD exhibited a significantly higher ROS level than DF at the basal level and 3 h and 12 h during low salinity exposure (Fig. 7D).

### Long-term effects of salinity on survival and growth of abalone

**Fig. 8:**
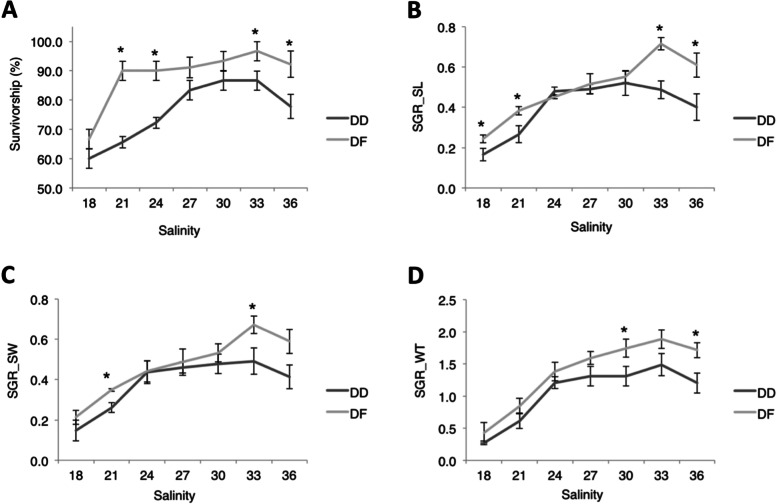
Survival and growth of *Haliotis discus hannai* (DD) and hybrid *H. discus hannai* ♀ × *H. fulgens* ♂ (DF) cultured under various salinities for 60 days: **A** Survivorship, **B** Specific growth rate in shell length (SGR_SL), **C** Specific growth rate in shell width (SGR_SW), and **D** Specific growth rate in wet weight (SGR_WT). Analysis of the differences between the species at a given salinity level was done by Welch’s t-test “*”: Significant difference between DD and DF at *P* < *0.05*

Salinity significantly (*P* < *0.05*) affected survival and growth in both species during long-term culture, indicating a reduction in both parameters at sub-optimal salinities (Fig. 8) Species differences were also observed, and suggested a significantly higher survival in DF at salinities of 21 (*P* = *0.001*), 24 (*P* = *0.003*), 33 (*P* = *0.02*), and 36 (*P* = *0.01*). Furthermore, specific growth rate in shell length (SGR_SL) was significantly higher in DF at 18 (*P* = *0.04*), 21 (*P* = *0.02*), 33 (*P* = *0.003*), and 36 (*P* = *0.02*), while specific growth rate in shell width (SGR_SW) was only significantly higher in DF at salinities of 21 (*P* = *0.04*), and 33 (*P* = *0.01*). Also, specific growth rate in wet weight (SGR_WT) was only significantly higher in DF at salinities of 30 (*P* = *0.03*), and 36 (*P* = *0.02*).

## Discussion

### The fluid shear stress and atherosclerosis (FSS) pathway

According to the Kyoto Encyclopedia of Gene and Genome (KEGG) pathway enrichment analysis, several pathways were considerably altered following low salinity exposure in the present study. Of particular interest was the fluid shear stress (FSS) and atherosclerosis pathway, for reasons like the absence of any published report on the FSS pathway in abalone. Meanwhile, the FSS pathway was in the top twenty significantly enriched pathways during low salinity exposure. Furthermore, a comparison between the control groups of both species revealed that the FSS pathway was not among the top 20 enriched pathways, which suggests that this pathway is triggered under hypo-osmotic stress. Also, some essential genes involved in this pathway are less studied in abalone, especially under salinity stress.

Furthermore, the FSS pathway is well studied in humans concerning cardiovascular diseases [31, 32]. Shear stress implies the frictional force that blood flow wields on the endothelial surfaces constantly exposed to it. Comparably, abalone gill ECs are constantly exposed to hemolymph flow [33], and available studies indicate that hemocytes that circulate in hemolymph and tissue sinuses of marine gastropods experience reduced propulsion and sluggish spread under salinity stress [34]. Hence, we speculated that abalone gill ECs could experience shear stress as observed in human ECs exposed to constant blood flow [35].

Therefore, this study delved into the FSS pathway with the hypothesis that abalone up-regulates the genes that foster anti-apoptosis and anti-inflammation during short-term salinity stress and down-regulates genes that promote inflammation and apoptosis. Consequently the gill epithelial cells (ECs) are protected, and their proper functioning by allowing the stable flow of well-oxygenated hemolymph to the heart and all parts of the abalone’s body is ensured.

### The expression patterns of the different FSS pathway genes in abalone

Heat shock proteins (HSPs) are a group of famous and highly conserved molecular chaperones engaged in numerous roles of the cellular stress response and are used as biomarkers of environmental variations [24]. Characterized by their molecular weight, HSPs include HSP90, HSP70, HSP60 and HSP20-30, and are the essential genes associated with environmental stress tolerance and the maintenance of cellular homeostasis in most marine organisms [36, 37]. HSP90 is a chief intracellular chaperone protein that guarantees accurate protein configuration and is observed in standard and stressed organisms. Furthermore, it protects organisms against stress by amending the wrong folding of the denatured proteins and also helps clear cells of denatured isoforms by proteolytic annihilation via the proteasome [38]. Additionally, HSP90 participates in immune response and protection against biotic and abiotic stress in some marine mollusks [33, 39, 40].

Available studies suggest that the level of HSP90 expression varied between tissues, organisms, and seasons and was notably regulated by cadmium exposure, bacterial infection, and fluctuations in temperature and salinity [41, 42]. For example, in the bay scallop, *Argopecten irradians*, HSP90 expression in the hemocyte was up-regulated 9 h following inoculation of bacteria and then declined to control levels at 48 h [39]. In the marine crab, *Portunus trituberculatus*, Zhang et al. [43] observed that HSP90 expression in the gills did not significantly change under low and high salinity stresses. Palmisano et al. [42] also recorded no induction of HSP90 in the gills of the Chinook salmon 24 h after a seawater challenge. Meanwhile, Pan et al. [44] noted a significant up-regulation of HSP90 in the branchial lamellae of the Atlantic salmon following 24 h of hyper-osmotic treatment, informing the conclusion that HSPs are crucial factors for acclimatization of salmon to hyper-osmotic stress. In the abalone *H. tuberculata*, up-regulation of HSP90 was significant within 30 min after heat shock [41]. Moreover, when the Pacific abalone was subjected to temperature treatment, HSP90 expression in the gills was significantly up-regulated after 12 h at 24 °C and 28 °C but was down-regulated only in the latter treatment after 96 h [45]. A similar HSP90 up-regulation expression was also detected when the disk abalone was exposed to heat shock [46], and Huang et al. [47] likewise confirmed a significant up-regulation of HSP90 in the gills of *H. diversicolor* following temperature and hypoxia treatments.

In the present study, low salinity significantly regulated the expression of HSP90 and proved to be time-dependent, as was also observed by Park et al. [45]. While no significant difference was observed at 3 h, relative to the control, it was significantly up-regulated in DF at 12 h and 24 h but down-regulated in DD after 3 h. It is speculated that immune cells produce reactive oxygen species (ROS) during low salinity stress, which could cause damage to the host cell and subsequent protein denaturation. The synthesis of HSP90 presumably fosters the renaturation and reassembly of the damaged proteins, unlike HSP70, which safeguards proteins against damage [42, 45]. Also, HSP90 proteins are principally anti-apoptotic [48]. Our study argues that HSP90 can offset the adverse changes caused by low salinity, and its down-regulation would impair the proper function of the abalone gill ECs. Available data from other marine invertebrates suggest the presence of more than one HSP90, which plays different roles in physiological and stressful conditions [43], but whether the same is true for abalone remains to be investigated.

Calmodulin-4 (CaM-4) is a highly conserved, multifunctional calcium regulatory protein that plays a crucial role in intracellular transduction [49–54]. Although most invertebrates reportedly have only one CaM gene, studies have revealed four non-allelic CaM genes in the Pacific abalone [55]. Evolving evidence on mollusks CaM gene suggests their involvement in calcium metabolism [55] and shell formation [54]. CaM is also known to bind to and regulate numerous proteins associated with inflammation and immune response [56]. In the fungus (*Sporothrix schenckii*), calmodulin kinases (CaMK) have been implicated in their temperature tolerance [57]. In some plants, down-regulation of CaM expression augmented vulnerability to infectious bacteria and fungi, but its overexpression conferred more excellent resistance to pathogens [58]. Calmodulin has also been studied in shrimps and sea cucumber under pathogenic infection [59, 60], in oysters under ocean acidification and temperature [61], and in fish, mussels, and crabs under salinity and pH stresses [26, 28]. These earlier studies suggest that CaM plays a central role in biotic and abiotic stress adaption and invertebrate innate immunity.

In 2014, S. Li et al. [62] and E. Li et al. [26] demonstrated that salinity stress significantly up-regulated CaM expression in the gill of the Chinese mitten crab in a time-dependent manner. Nikapitiya and Lee [63] also observed a significant up-regulation of CaM expression in the gills within 3 h after bacterial challenge in the disk abalone. Similarly, CaM expression in the present study was significantly up-regulated at 3 h and reached the highest at 12 h. In addition, an increase in CaM expression has been observed in tissues related to osmoregulation and metabolism in many marine invertebrates. For instance, S. Li et al. [62] observed that the gill was among the tissues with the highest expression of CaM and hypothesized that the gene might be involved in ion balance. Lim et al. [55] also observed that all the four characterized CaM genes of the Pacific abalone were expressed in the epithelial tissues (gill and mantle), which are involved in direct calcium uptake. Likewise, under salinity stress, gill CaM was significantly up-regulated in the blue mussels [28].

The high expression and sustained stimulation of calcium-regulatory proteins during the present salinity challenge, as was observed in the gill and mantle of the disk abalone [64], submit that these proteins are required to regulate Ca^2+^, and that robust transcription is necessary for their synthesis. Meanwhile, S. Li et al. [62] theorized that continuous high expression of CaM could excessively stimulate the ion channels and result in damaging effects; hence, CaM expression rose and dropped again with time. This two-phase expression pattern was also noticed in the present study. Our data, thus, insinuate that the abalone CaM gene might as well be necessary for maintaining ion equilibrium by controlling the actions of stress-related ion conduits and is a crucial gene mediating abalone innate immunity. Consistently, Nikapitiya et al. [64] linked the upkeep of calcium homeostasis by calcium-regulatory protein with the immune-defense mechanism in the disk abalone. Furthermore, Ji et al. [59] also associated the observed significant down-regulation of CaM expression, following pathogen injection, to a weakened defense against such infection. Moreover, studies suggest that the role of CaM in thermotolerance could be via its influence on HSP90 [57], which could also be true for its role in salinity tolerance in abalone.

Tumor necrosis factor (TNF) is a group of proteins belonging to the TNF superfamily [65] and can modulate many cellular processes, including inflammation, apoptosis, and phagocytosis [21, 66]. Studies on invertebrate’s TNF superfamily have been conducted in abalones, sea squirts, oysters, and scallops, under pathogen infection, and implicated in their immune responses [20, 67–69].

Existing literature also submits that the influences of TNF on fundamental cellular processes are specific in various contexts. For instance, Sun et al. [65] demonstrated that TNF significantly induced apoptosis and phagocytosis of the oyster *Crassostrea gigas* hemocytes. Contrarily, TNF considerably induced proliferation and inhibited apoptosis of human nucleus pulposus (NP) cells [70]. Moreover, TNF was significantly up-regulated during pathogen infection in the disk abalone’s (*Haliotis discus discus*) gill after 3 h, 12 h, and 24 h [21].

However, in the present study, TNF was significantly down-regulated during low salinity stress throughout the observed experimental period. Yu et al. [69] noted that the release of pro-inflammatory cytokines like TNF might either be advantageous or damaging to the host subject to the circumstance of its production. Our data, thus, suggest that abalones could significantly prohibit inflammations or apoptosis and consequent cell damage during short-term salinity stress by down-regulating the expression of TNF.

Nuclear factor-kappa B (NF-kB) is a stimuli-triggered transcription factor that regulates many downstream genes involved in survival, inflammation, development, apoptosis, and immune response of many cell types [71]. NF-kB transcripts have previously been isolated from mollusks, including oysters, abalones, and scallops [71–73], and showed a vital role in their innate immune response during pathogen challenges.

In the scallop *Chlamys farreri*, NF-kB was markedly up-regulated in a time-dependent manner during lipopolysaccharide (LPS) stimulation [73]. Likewise, a significant up-regulation of NF-kB was observed in the hemocyte of the disk abalone *H. discus discus* following bacterial challenge [74]. Apart from the induction of NF-kB signaling by toll-like receptor 4 (TLR4) during the recognition of pathogens and by TNF, several studies have demonstrated that abiotic stresses also activate the NF-kB signaling cascade due to oxidative stress and injury [75, 76]. Earlier studies have also confirmed that elevated NF-kB activation is associated with inflammation [77].

In the present study, NF-kB was significantly down-regulated 3 h after low salinity challenge and was significantly up-regulated after 24 h. The expression profile, thus, infers that prolonged exposure of abalone to low salinity could induce inflammations on the gill epithelial cells. Our result could be likened to what Zhang et al. [78] observed in the small abalone *H. diversicolor* under temperature and hypoxia exposure. In their study, NF-kB was up-regulated significantly in gills only after 24 h.

Moreover, it is hypothesized that NF-kB activation and the subsequent induction of pro-inflammatory cytokines during pathogen infection are essential to clear the invading bacteria [79]. Thus, it makes sense that in the absence of pathogens such as in the present study, the initial response is a marked down-regulation to protect the host cells. However, any observed latter up-regulation infers a time-dependent build-up of oxidative stress in the host cells, which would induce inflammation and cell damage. Furthermore, our data confirm that the regulation mechanism of NF-kB might differ under biotic and abiotic stresses, as was also observed in the mud crab *Scylla papamamosain* by Jiang et al. [75].

Additionally, the transcripts of the inhibitor of NF-kB kinase (IKK), which act upstream of NF-kB, showed different expression profiles against various pathogens and submitted that crabs had differing immune-response mechanisms against diverse pathogen toxicity [75]. Again, Jiang and co [75] noted that the IKK transcripts showed distinct expression patterns in various tissues under air exposure, which they assumed that IKK’s response to stress might be tissue-specific. These observations again lead us to suspect that NF-kB expression in abalone might differ under various stresses and could be tissue-specific under the same abiotic stress. Hence, further study would be necessary to understand the potential role of NF-kB in initiating and controlling the innate immune system of abalone under abiotic stresses.

Bone morphogenetic proteins (BMPs) belong to the transforming growth factor-β (TGF- β) superfamily and are known to control a wide array of biological processes, including cell proliferation and apoptosis in myriad cell types [80, 81]. An et al. [82] testify that BMP-4, a member of the BMP family, plays a vital role in energy metabolism. In humans, BMP-4 is pro-inflammatory and can stimulate oxidative stress in cardiovascular cells [35]. Also, there is evidence that BMP-4 could directly stimulate ROS overproduction, which can induce oxidative stress and result in perpetual cell damage [83]. Furthermore, BMP-4 sparked ROS overproduction and led to EC apoptosis via the p38/JNK pathway in some vertebrates [80]. Besides, apoptosis is one of the cellular functions targeted throughout osmotic stress in fish gill [84]. Meanwhile, the down-regulation of BMP-4 aids anti-inflammatory effects [83].

Furthermore, Park et al. [45] confirmed that accrued intracellular ROS would disturb the functions of particular tissues and organs, but more importantly, will cause premature death of the entire organism. Oxidative stress is also involved in many biological and pathological processes, such as inflammation. Data from the present study signifies that abalones significantly down-regulated BMP-4 to prevent inflammations, apoptosis, and subsequent gill EC dysfunction during short-term salinity stress.

### Potential crosstalk between the different signaling cascades

In Fig. 4B, a hypothesized model of the FSS pathway in abalone shows some genes and the potential crosstalk between the signaling cascades. In line with this hypothesized model, we believe that low salinity altered the intracellular Ca^2+^ of abalone and therefore triggered CaM-4. Consequently, CaM-4 induced HSP90 expression, and the increased expression of HSP90 assisted the repair of oxidized CaM-4, thus, its sustained up-regulation. Furthermore, we believe that low salinity altered the abalone’s cytosolic Ca^2+^, which activated the MAPK signaling directly or through the activation of TNF, and consistently triggered the NF-kB signaling downstream. Moreover, we believe that the BMP signaling was directly triggered by low salinity stress or through TNF activation. Also, transmembrane proteins such as p67^phox^ and p22^phox^ could exacerbate BMP4-induced ROS production in abalone gills under prolonged exposure to low salinity.

From our data, the expression profiles of CaM-4 and HSP90, especially in the hybrid DF, suggest that CaM-4 interacts with HSP90 to confer salinity tolerance in abalone as was hypothesized in yeast thermotolerance [57]. Calcium is a central intracellular secondary messenger that can influence many cellular processes by binding to calmodulin, a well-known prime sensor of Ca^2+^ signals [57, 85]. In addition, circumstances such as bacterial infection, high temperature, and low salinity, which trigger oxidative stress, also alter the intracellular Ca^2+^ [58, 59, 83], and the consequent variation in cytosolic calcium activates the CaM-dependent kinases [62].

CaM, upon Ca^2+^ binding, adopts an open conformation that allows its regulation of a range of downstream target proteins in a Ca^2+^-dependent way, which is consistent with its observed regulation and interaction with the heat shock protein 90 (HSP90) to confer thermotolerance [57]. Hence, we hypothesize that low salinity would alter the abalone intracellular calcium and thus induce CaM, increasing HSP90 expression. Furthermore, Rodriguez-Caban et al. [57] observed that decreasing the levels of CaM yielded cells intolerant to high temperatures by affecting the function of HSP90.

Also, oxidative stress situations result in selective oxidative alteration on calcium regulatory proteins like CaM, causing a decline in their ability to trigger an array of diverse proteins [50]. HSP90, whose expression is up-regulated under oxidative stress, has also been implicated in assisting the repair or degradation of oxidized CaM via selective recognition; thus, restoring cell function. Again, we hypothesize a feedback loop in this study, where the HSP90, triggered in a CaM-dependent manner, would mediate the recognition and subsequent repair or degradation of oxidized CaM, thus, sustaining the physiological mechanism of adaptive cellular response to oxidative stress. Rodriguez-Caban et al. [57] again proposed an interaction whereby CaM regulates HSP90, and HSP90, in turn, regulates CaM to aid thermotolerance in yeast.

Secondly, oxidative stress from either biotic or abiotic stimuli induces pro-inflammatory cytokines such as TNF. Consequently, NF-kB is activated in response to these cytokines [79]. In the present study, relative to the control, the down-regulation of TNF at 3 h coincided with the relative down-regulation of NF-kB, while a rise in TNF expression at 12 h saw a consequent rise in NF-kB. However, the expression of NF-kB was significantly up-regulated than TNF at all the observed experimental times, indicating that the relative rise in TNF expression nearly doubled NF-kB expression. The data confirm that the NF-kB pathway is activated by pro-inflammatory cytokines, as observed in some mollusks [79].

Nuclear factor-kappa B (NF-kB) and TNF are key signaling cascades implicated in the FSS pathway [31]. In mammalian cells, elevated cytoplasmic Ca^2+^ supposedly activates NF-kB [86]. Notably, the innate immune response of invertebrates activates cellular activities primarily through NF-kB [75]. Also, TNF is a crucial cytokine involved in many processes in vertebrates and invertebrates [65]. Available literature suggests crosstalk between human TNF, NF-kB, c-Jun N-terminal kinase (JNK), and p38 MAPK [70]. Yang et al. [87] also give an account of how the activation of TNF**-**α leads to downstream signaling pathways that digress to IKK and JNK activation through discrete MAPK kinase kinases (MEKK). Moreover, TNF reportedly induced the stress-activated p38 and JNK MAPKs in the bivalve *Mytilus galloprovincialis* [88]. Thus, TNF interchangeably induces cell survival/inflammation or apoptosis [77]. Moreover, Park et al. [68] suggest that NF-kB regulates TNF transcription. We, thus, theorize that such crosstalk could exist in the abalone as well.

In another school of thought, however, the expression of NF-kB could be signaled via a different pathway than the theorized TNF/NF-kB signaling pathway, as noted in some literature. For instance, Wang et al. [70] speculated that distinctive pathways might be involved in TNF effects in different cell types. Likewise, Anderson et al. [89] suggest an alternate mechanism for IKK and NF-kB activation, directly involving the receptor-interacting protein (RIP) or RIP through the MEKK intermediate. Mitogen-activated protein kinase (MAPK) signaling cascades play a principal role in transducing several signals in organisms, and are triggered by diverse external stimuli, including osmotic shock [90]. For example, Sun et al. [91] noted that activated MAPKs induced the nuclear factor-kappa B (NF-kB) signaling downstream in some vertebrates. Also, in the disk abalone, *H. discus discus*, NF-kB reportedly was not involved in regulating TNF expression during bacterial and virus challenge [20].

Regulation of TNF by NF-kB is known to protect cells from TNF-induced apoptosis [92]. Our data could speculate that under short-term salinity challenge, NF-kB regulates TNF to induce cell survival or inflammation and prohibit TNF-induced apoptosis. Further studies would be necessary to understand better the crosstalk between TNF and NF-kB signaling pathways in abalone.

Studies on bone morphogenetic proteins (BMPs) have heightened since they were discovered to play a crucial role in the regulatory function of various tissues and organs apart from their involvement in bone formation [35]. Again, Luo and co [35] reported that BMP signaling is regulated upstream by oscillatory shear stress (OSS) and pro-inflammatory stimuli such as TNF, resulting in the up-regulation in endothelial BMP4 expression. Also, there is a theorized link between reactive oxygen species (ROS) stimulation, OSS-induced BMP4, and EC inflammation [35]. Specifically, transmembrane proteins including p67^phox^ and p22^phox^ are triggered by pro-inflammatory stimuli, which exacerbate BMP4-induced ROS production [35]. Additionally, transcriptome analysis of the Sydney rock oyster *Saccostrea glomerata* revealed the involvement of p22^phox^ and p67^phox^ in ROS production [93]. Thus, in the present study, we hypothesize that low salinity could induce EC inflammation via up-regulation of BMP4. Likewise, some published data suggest crosstalk between BMPs, TNF, and NF-kB [35, 71].

Other genes that are involved in the FSS pathway [30], some of which were also expressed in the abalone in the present study (Fig. 4B), include phosphatidylinositol 3-kinase (P13-K), B-catenin, AMP-activated protein kinases (AMPK), and some mitogen-activated protein kinases (MAPKs). Blanc et al. [94] suggest phosphatidylinositol 3-kinase (P13-K) catalyzes the production of phosphatidylinositol 3,4,5-triphosphate (PIP3), which phosphorylates the serine/threonine kinase PKB/Akt in threonine (Thr^308^). Akt is reportedly a vital player in many physiological processes, including cell survival and death. In the small abalone, *H. diversicolor*, PI3K-Akt was one of the immune-related pathways regulated during hypoxia stress [95]. Moreover, hydrogen peroxide (H_2_O_2_), which is produced during the process of superoxide anion (O_2_^−^) dismutation, is assumed to trigger both p38 MAPK and PKB signaling systems in a Ca^2+^—and CaM-dependent manner [94]. Furthermore, alterations in cytosolic Ca^2+^ concentration reportedly activated MAPKs [51, 58, 85]. Again, it could be hypothesized that low salinity induced oxidative stress, leading to the activation of these signaling systems.

While available data establishes that H_2_O_2_ can impact Ca^2+^ homeostasis in myriad cell types, studies in humans have shown that slight intracellular Ca^2+^ elevation could activate Akt via CaM-dependent protein kinase kinase (CaM-KK), an upstream kinase of Akt, thus inhibiting apoptosis in cells [49]. Likewise, the Kruppel-like factor (KLF) expression in abalone hemocyte is implicated in the cellular immune pathway during bacteria challenge [29].

Hypothetically, nitric oxide (NO) acts downstream of Ca^2+^ signals by the action of CaM to induce the hypersensitive response (HR) against pathogens in plants [58]. Similarly, CaM is vital for NO production in invertebrate echinoderms [96]. Furthermore, NO controls chloride cell function in fish gills in reaction to osmotic stress [27]. Tumor necrosis factors (TNFs) are also noted to induce nitric oxide to regulate the innate immune response in oysters [66] and were found to suppress the expression of endothelial nitric-oxide synthase (eNOS), increase ROS generation, and consequently reduce NO levels in humans [89]. Although it is a vital gaseous signaling molecule involved in immune response, NO could react with ROS to produce extremely powerful oxidant peroxynitrite (ONOO^−^), which increases toxicity and inhibits DNA repairs [66].

Altogether, we could assume that activating genes such as CaM-4 and HSP90 could enhance anti-inflammation, anti-oxidation, and anti-apoptosis. On the other hand, activating genes such as TNF, BMP4, and NF-kB could augment apoptosis and inflammation, as illustrated in Fig. 4B.

### The possible connection between molecular, cellular, and phenotype data

The process of phagocytosis is an immune intervention that is also triggered by abiotic factors and is accompanied by ROS production [97]. Our analysis of abalone hemolymph by the flow cytometer confirms a significantly increased phagocytosis at 3 h, 12 h, and 24 h. Consequently, ROS production increased significantly at 12 h and 24 h. Altogether, our data hints that ROS begins to build up in abalone tissues after 3 h under low salinity, which could result in oxidative stress and a subsequent rise in the expression of pro-inflammatory genes. Consistently, it could be observed that expression of all genes that aid pro-inflammation and apoptosis began to rise gradually after 3 h. Similarly, a significant impact of salinity was noted in the Pacific abalone by 3 h after salinity variation, which led Jai and Liu [6] to resolve that 3–6 h is the precarious time scope. However, reports from other marine invertebrates suggest that low salinity reduced phagocytosis and influenced phagocytic activity in a species-specific manner [98]. Meanwhile, increased ROS production in response to salinity stress has been confirmed in other abalone species [34]. Furthermore, previous data submit that abalone has a non-specific innate immune defense system at a basal level to combat raiding pathogens [16], consistent with the phagocytic activity and ROS in the control groups of the current study.

Decreased total hemocyte count (THC), as was observed in the current study during low salinity exposure, is supposedly due to a relocation of hemocytes to the adjacent tissues that might be predisposed to injury [99]. Several reports have demonstrated that fluctuations in salinity could decrease the resistance of some marine invertebrates to pathogens due to the consequent decline in THC [100, 101]. Furthermore, hemocyte mortality increased during low salinity exposure, which has also been associated with increased oxygen free radicals induced by stress factors. In 2019, Yang and Min [9] also observed a decline in THC and a rise in hemocyte mortality when *H. discus hannai* was subjected to low salinity stress. Likewise, low salinity decreased hemocyte number in mussels and crabs [98, 102, 103]. Because hemocytes are necessary for synthesizing osmotic shock protein that confers protection from acute salinity changes, as reported in some bivalves [98], a decline in THC could threaten abalone’s well-being during prolonged exposure to low salinity.

Like a heat-shock response, osmoregulation is energetically costly. Under sub-optimal salinity conditions, abalone is likely to reduce feeding or divert the energy for growth and development to osmoregulation and other adaptive physiological functions that aid survival. Likewise, Li et al. [104] observed that organisms would frequently repress energy production for growth to ensure the sustenance of life during stressed conditions. In the current study, we see a reduced growth rate of abalone after 60 days at low salinities. Our data is consistent with that of Cheng et al. [105], who also observed a negative correlation between growth rate and the expression of molecular chaperones in abalone under stressful environmental conditions.

### Interspecies differences in molecular, cellular, and phenotypic responses

Both species showed changes in the expression of the reported genes involved in the FSS pathway, signifying the importance of the pathway in osmotic stress response in abalone. However, there were notable species-specific differences in the expression of some of these genes over the observed experiment time.

Firstly, HSP90 was significantly up-regulated in DF throughout the treatment period compared to DD. In 2019, Chen et al. [22] demonstrated that the heat-tolerant abalone line showed a more remarkable average fold change in the expression of HSP genes than the heat-sensitive abalone line. Similarly, Cheng et al. [105] observed a higher expression of HSP70 in hybrid abalone than inbred abalone at high temperatures close to the upper physiological tolerable limit, which they ascribed to the capacity of the hybrid population to buffer injury triggered by elevated temperatures in the cell. In 2017, Yan and co [106] confirmed that hybrid oysters showed higher expression of immune-related genes and genes involved in osmoregulation than their purebred counterparts. We could assume from the pattern of HSP90 expression in the Pacific abalone and the hybrid DF that the latter might have a more effective response tactic to low salinity, hence, a better tolerance for low salinity than the former. The up-regulation of HSP90 is hypothesized to regulate the cell’s cytosolic redox condition for defense against oxidative stress, thus, activating a long-term protective mechanism [45].

Furthermore, Zhang et al. [43] hypothesized that the marked down-regulation of HSP90 in some tissues, following osmotic stresses, was symbolic of the surpassed tolerance boundaries leading to cell death. Meanwhile, tissues with better tolerance exhibited significantly up-regulated HSP90 expression. The marked reduction in HSP90 expression after 3 h in DD could be attributed to the postulated negative regulation mechanism, where the expression levels of HSPs decline after attaining a high abundance [45]. Our data suggest that the Pacific abalone could be more prone to severe low salinity stress after 3 h of exposure.

In the Pearl oyster, *Pinctada fucata*, Liu et al. [61] hypothesized that the time-dependent decrease in the expression of HSP70 under heat shock was associated with diminished energy budget to meet the energy demand for HSP70 synthesis. Similarly, we could assume that the significant down-regulation in the expression of DD’s HSP90 at 12 h and 24 h could be due to surpassed tolerance boundaries and diminished energy budget, contrary to what was observed in the hybrid DF. Such an assumption would be consistent with our earlier data [15] on the heart rates of the two species under hypo-osmotic stress, where the performance of the hybrid DF suggested a better metabolic activity and energy balance than DD. In addition, Shen et al. [107] also demonstrated how the hybrid DF is a better metabolic modulator under hypoxia treatment.

Moreover, in the Pacific abalone, sustained up-regulation of CaM-4 did not result in sustained up-regulation of HSP90, implying a failure in the proposed feedback loop postulated earlier on. However, existing findings suggest inter and intra-species variation in the genes coding for CaM [59]. In addition, interspecific differences in gene expression in response to an acute decrease in salinity were observed in two blue mussels species [28].

Regarding cellular immune response, the significantly high basal hemocyte mortality and ROS in the Pacific abalone indicate that the species might have been undergoing some other stress though both DD and DF were acclimatized under the same environmental conditions. It might also propose the general sensitivity of DD to environmental stresses. Meanwhile, a previous study shows the increased level of abnormal proteins in inbred abalone, which may spark persistent cellular stress from an attempt to restore protein homeostasis even under benign environmental conditions [105]. However, the observed comparative profiles of DF and DD insinuate a more active hemocyte-mediated immune response in DF under salinity stress. For instance, in DF, phagocytosis was significantly high at 3 h and 24 h, ROS was significantly low at 3 h and 12 h, and hemocyte mortality was significantly low at 3 h and 24 h. Similarly, in two species of abalone, Martello et al. [34] observed that the more salinity-sensitive one recorded significantly lower % phagocytosis during the osmotic challenge.

Furthermore, DF exhibited superiority in survival and some growth parameters like shell length and shell width, which was significant at a low salinity of 21. This data on cellular response and expression of some genes revealed some candidate mechanisms driving the phenotype difference in survival and growth between the species at sub-optimal salinities. Also, DF’s performance on survival and growth at both low and optimal salinities suggests somewhat phenotypic plasticity as described in the literature [108, 109].

Taken together, we could assume that both inbred and hybrid abalone elicits immune responses via similar mechanisms under low salinity stress. However, the hybrid DF demonstrates a more efficient capacity, which aids its higher survival. To some extent, the data on transcriptomics complements that on the cellular immune response and growth and survival between the two species. However, it would be necessary to elucidate their response to salinity stress at the proteome level since the physiological mechanism in response to environmental stress goes beyond the level of gene expression, as hypothesized by Lockwood and Somero [28].

From previously reported studies, we understand how abalone adapts to environmental salinity changes by maintaining their osmotic pressure balance, which is achieved via regulation of their intracellular ions, hemolymph free amino acids, and changes in ion-regulatory genes expression [6–8]. Furthermore, abalone achieves salinity adaptation through modulation of the heart rate by adjusting oxygen consumption and respiratory metabolism [5, 15]. Moreover, immune defense is accomplished via the regulation of antioxidant genes and the employment of hemolymph parameters like phagocytosis and respiratory burst [7, 9]. In addition, the present study highlights how abalone engages the FSS pathway and related genes in dealing with low salinity exposure. Therefore, we can anticipate that abalone adapts several mechanisms to survive dire salinity situations.

## Conclusion

Our data highlights the molecular mechanism by which abalone responds to low salinity. Our study shows that abalone survives short-term low salinity stress by up-regulating the genes that promote anti-oxidation and anti-inflammation, such as CaM-4 and HSP90 and down-regulates the genes that promote inflammation and apoptosis such as TNF, NF-kB, and BMP-4. The present study also explains how these essential proteins and molecular mechanisms collaborate in the FSS pathway to bestow tolerance to osmotic stress in abalone. Meanwhile, long-term exposure of abalone to low salinity could result in damaged gill ECs due to inflammations or apoptosis; hence, disturbed flow of well-oxygenated hemolymph to the heart and other essential organs. Finally, the data confirms that hybridization could be a method for breeding more stress-resilient aquatic species.

## Materials and methods

### Experiment setup and short-term low salinity exposure

Pacific abalone *H. discus hannai* (herein referred to as DD, 30 individuals, 60.2 ± 1.5 mm shell length) and hybrid *H. discus hannai* ♀ x *H. fulgens* ♂ (herein referred to as DF, 30 individuals, 60.4 ± 1.6 mm shell length) were collected from Fuda abalone farm (Jinjiang, China) and transported to the experiment site in Xiamen University. The abalones were then randomly distributed into rectangular containers in triplicates (36 cm × 20 cm × 20 cm; ≈ 25 L water holding capacity and equipped with aerators; 10 abalones per container) and acclimatized for a week. Throughout the acclimation, dissolved oxygen concentration, pH, and nitrite were monitored daily and remained at the appropriate levels of 8.0 ± 0.2 mg.l^−1^, 8.2 ± 0.2, and 0.001 ± 0.1 mg.l^−1^, respectively. Also, observed temperature and salinity were 24.6 ± 0.8 ℃ and 33, respectively. Nitrite was measured with a handheld colorimeter (Hanna Instruments, HI764), and temperature, dissolved oxygen, and salinity were measured with a handheld water quality meter (86,031 AZ Waterproof IP67 Combo Water Quality Tester). Furthermore, abalones were fed with red algae (*Gracilaria lemaneiformis*) and experiment water was changed daily.

Following acclimation, salinity was gradually reduced from 33 to 21 at a rate of 4 levels per hour, and abalones were maintained at the salinity of 21 until sampling. Meanwhile, salinity reduction involved adding fresh tap water (de-chlorinated by storage in a clean tank for not less than three days, under constant aeration) to freshly filtered seawater to the desired salinity and then transferring into the abalone containers.

### Collection of samples and analysis

Before the salinity variation, 12 abalones (2 individuals × 3 replicates × 2 species) were randomly selected as control (0 h). Also, after the salinity variation, 36 abalones were randomly selected at three hours, 12 h, and 24 h (2 individuals × 3 replicates × 3 sampling time × 2 species).

#### Hemolymph collection and flow cytometry analysis

Approximately 2 mL of hemoplymph was collected from each abalone in control and treatment groups. First, the middle of the foot muscle was cleansed with gauze and absorbent cotton, followed by a longitudinal incision with a pasteurized blade. Subsequently, hemolymph was drawn with a pipette into a 5 mL centrifuge tube and stored on ice until flow cytometric analysis. All flow cytometric analyses, including total hemocyte count (THC), hemocyte mortality, phagocytosis, and reactive oxygen species (ROS) production, were successively done after the protocol described by Shen et al. [18].

#### Gill tissue collection and transcriptome analysis

Immediately after hemolymph collection, each abalone was shucked and both the right and left gill tissues were collected into separate cryogenic tubes. Samples were then instantly snap-frozen with liquid nitrogen and stored at -80 ℃ for subsequent RNA extraction. For each species, twelve biological replicates, including four individuals from the control group, four from the treatment group at three hours, and four from the treatment group at 24 h, were used for transcriptome analysis.

Total RNA extraction, library construction, and sequencing: Novogene Bioinformatics Technology Co., Ltd carried out RNA library construction and sequencing. Briefly, total RNA was extracted from gill tissues by the TRIzol method (Invitrogen, USA). Subsequently, Qubit® RNA Assay Kit in Qubit® 2.0 Fluorometer (Life Technologies, CA, USA) and the RNA Nano 6000 Assay Kit of Agilent 2100 Bioanalyzer system (Agilent Technologies, CA, USA) were used in assessing the RNA quantity and integrity, respectively.

Following the RNA extraction, random hexamers primer and reverse transcriptase were used to synthesize cDNA from the mRNA template. After first-strand synthesis, a custom second-strand synthesis buffer (NEBNext® Ultra™ RNA Library Prep Kit for Illuminia®; NEB, USA) was added with dNTPs, RNaseH and Escherichia coli polymerase I to generate the second strand by nick-translation. Afterwards, purification, terminal repair, A-tailing, ligation of sequencing adapters, size selection and PCR were performed. Finally, Qubit® 2.0 Fluorometer (Life Technologies, CA, USA) was used to check library concentration, and Agilent 2100 Bioanalyzer system (Agilent Technologies, CA, USA) was used to check library quality. Libraries were then sequenced on the Illumina X ten platform, generating 150 bp paired-end reads.

Raw data cleaning and alignment: The raw data was filtered to remove connector adapter, N bases, and low quality reads. After quality control, clean reads from 18 libraries were generated for subsequent bioinformatics analysis. Then the clean reads were aligned to the *Haliotis discus hannai* reference genome (unpublished data) using HISAT2 [110] software with the following parameters: ${HISAT2} -p ${THREADS} -x ${GENOMEIDX} -1 ${Reads1} -2 ${Reads2} -S ${Sample_sam}.

Gene expression quantification and differentially expressed genes identification: StringTie [110] software was used to estimate gene expression abundance in each sample with the following parameters: $STRINGTIE -e -B -p ${THREADS} -G ${GTFFILE} -A ${Sample_gene_abund} -o ${Sample_gtf} ${Sample_bam}. Reads Per Kilobases per Million reads (FPKM) method was then used to quantify the expression levels of genes from different samples. Furthermore, DESeq2 [111] software was used to identify the differentially expressed genes (DEGs) by comparisons between the control, 3 h, and 24 h (DD: CDvsD3, CDvsD24, D3vsD24; and DF: CFvsF3, CFvsF24, F3vsF24), and between the two species at control (CDvsCF), 3 h (D3vsF3), and 24 h (D24vsF24). Meanwhile, |log2(FoldChange)|> 1 & padj < 0.05 standard was adopted to screen the differential genes. Furthermore, Principal Component Analysis (PCA) was performed based on the FPKM values of all sample’s genes.

Functional enrichment and analysis: To understand the fundamental molecular mechanisms underpinning low salinity adaptation in abalone, we carried out functional enrichment analysis of the DEGs using clusterProfiler [112] software. The DEGs of each compared group were significantly enriched on KEGG (Kyoto Encyclopedia of Genes and Genomes) pathway with *p*-values < 0.05.

#### Experimental validation of FSS pathway genes by qRT-PCR

Gill tissues extracted from twenty-four biological replicates of each species, including six individuals from the control group, six from the treatment group at three hours, six from the treatment at 12 h, and six from the treatment group at 24 h, were used for differential expression verification by qRT-PCR. Subsequently, the expression profiles of 5 genes that are involved in the Fluid shear stress (FSS) and atherosclerosis pathway including, calmodulin-4 (CaM-4), heat shock protein90 (HSP90), tumor necrosis factor (TNF), bone morphogenetic protein-4 (BMP-4), and nuclear factor kappa-B (NF-kB) were verified.

Briefly, total RNA was extracted from the abalone gills using the Trizol method, following the manufacturer’s guidelines. NanoDrop 2000 spectrophotometer (Thermo Scientific) was used to assess the purity and the concentration of the extracted RNA by measuring the absorbance at 260 nm and 280 nm, while the Agilent 2100 Bioanalyzer system was used to assess the RNA integrity. Purified RNA was diluted to 1000 ng/μL, and one microliter of RNA from each sample was reverse-transcribed into cDNA using the PrimeScript RT Reagent Kit (Takara, China) per the manufacturer protocol. Furthermore, Primer3web (version 4.1.0) online software was used to design gene-specific primers (Table 1). The relative abundance of mRNA levels was assessed in triplicates with the applied biosystems® QuantSudio™ 6 Flex Real-Time PCR system (Life Technologies Life, CA, USA) using Faststart Universal SYBR Green Master (Rox) (Roche). The PCR cycling protocols were: 40 cycles of 95 ℃ for 15 s and 57 ℃ for 30 s; followed by a melting curve of 57 ℃ for 1 min and 95 ℃ for 15 s. Finally, the 2 ^–ΔΔCT^ method was adopted to estimate the differential gene expression.

**Table 1:** Primer sequence of real-time PCR products of different genes with accession numbers

Gene accession no	Gene name	DD	DF
		Primer sequence (5’-3’)	Primer sequence (5’-3’)
HDH_T11838	CaM-4	F: CGACCATGAAGCACTGAAGG R: CTGTTTCAGCTCTGCGTGTT	F: GAACACGCAGAGCTGAAACA R: AGCGTCCTTCCTCTCTTCAC
HDH_T19534	HSP90	F: TCAGCCTACCTTGTTGCAGA R: CATGTAAAGGGTGATGCGGG	F: TCAGCCTACCTTGTTGCAGA R: CATGTAAAGGGTGATGCGGG
HDH_T17720	TNF	F: AAGGGATGGGAAAGAGGAGC R: GCCCGAGTCAGTTTTGGATC	F: CCGGTTAAATCATGGGCTGG R: CTTCTTCTTCCGCTGCTTCC
HDH_T20016	BMP-4	F: CATGCAAAGACACCCCAGAG R: CCAGCCCATGATTTAACCGG	F: CCGGTTAAATCATGGGCTGG R: CTTCTTCTTCCGCTGCTTCC
HDH_T10224	NF-kB	F: CAGCCTTCATGTACGCACTC R: GCTTGTGTGCGGAAGTAACA	F: ATGACAGCAGCTCCAGACTT R: AGCTTTAACCAGGTCCTCCC
Reference gene	β-actin	F: GGTATCCTCACCCTCAAGT R: GGGTCATCTTTTCACGGTTG	F: GGTATCCTCACCCTCAAGT R: GGGTCATCTTTTCACGGTTG

### Long-term culture of abalone under various salinities

In another experiment, *H. discus hannai* (DD; 16.0 ± 0.6 mm shell length; 11.6 ± 0.4 mm shell width; 0.6 ± 0.1 g wet weight) and hybrid *H. discus hannai* ♀ x *H. fulgens* ♂ (DF; 15.0 ± 0.2 mm shell length; 10.7 ± 0.2 mm shell width; 0.5 ± 0.1 g wet weight) were cultured at various salinities (36, 33 as control, 30, 27, 24, 21,18, and 15) for 60 days, following the experiment set-up described in our previous study [10]. Subsequently, survival rate and growth rate were estimated.

### Statistical analysis

The R software version 3.3.3 was used to analyze all data. Also, Principal component analysis (PCA) based on the whole gene expression profile was conducted using R and a PCA scatter plot was drawn using ggplot2 package [113]. One-way ANOVA was used to analyze the differences of gene expression and hemolymph parameters between the sampling times for each species, followed by Turkey’s HSD test where applicable. Also, Welch’s t-test was used to analyze differences in gene expression and hemolymph parameters between the two species at any given sampling time. Furthermore, Welch’s t-test was used to analyze differences in survival and growth between the two species at a given salinity level. Differences were deemed significant at *P* < *0.05* and extremely significant at *P* < *0.01*.

## Supplementary Information

**Additional file 1:** **Supplementary Figure 1.** Venn diagram of common and differentially expressed mRNAs of gill tissues of abalone during short-term low salinity exposure: *H. discus hannai *(DD) and hybrid *H. discus hannai* ♀ × *H. fulgens* ♂ (DF). A) Comparisons within DD (CDvsD3, CDvsD24, D3vsD24), B) Comparisons within DF (CFvsF3, CFvsF24, F3vsF24), and C) Comparisons between DD and DF (CDvsCF, D3vsF3, D24vsF24). Controls: CD, CF; 3 h at low salinity exposure: D3, F3; and 24 h at low salinity exposure: D24, F24. **Supplementary Fig. 2.** Volcano plots of the differentially expressed genes (DEGs) of gill tissues of abalone during short-term low salinity exposure: I) *H. discus hannai *(DD): A. Comparison in DD between control (CD) and low salinity after 3 h (D3), B. Comparison in DD between control (CD) and low salinity after 24 h (D24). II) Hybrid *H. discus hannai* ♀ × *H. fulgens* ♂ (DF). A. Comparison in DF between control (CF) and low salinity after 3 h (F3) B. Comparison in DF between control (CF) and low salinity after 24 h (D24). Red color denotes up-regulated genes and Blue color denotes and down-regulated. **Supplementary Fig. 3.** Comparison of top 20 Kyoto Encyclopedia of Gene and Genome (KEGG) pathways enrichment statistics of gill tissues of abalone during short-term low salinity exposure. *H. discus hannai *(DD) and hybrid *H.discus hannai* ♀ × *H. fulgens* ♂ (DF). FSS pathway is highlighted with a rectangular red box. (I) Enrichment analysis for DD: A. Comparison between control (CD) and low salinity after 3 h (D3), B. Comparison between control (CD) and low salinity after 24 h (D24), and C. Comparison between low salinity groups after 3 h and 24 h (CD3vsD24). (II) Enrichment analysis for DF: A. Comparison between control (CF) and low salinity group after 3 h (F3), B. Comparison between control (CF) and low salinity group after 24 h (F24), and C. Comparison between low salinity groups after 3 h and 24 h (F3vsF24). The size of each point represents the number of genes annotated to the KEGG pathway. Different colors from yellow to mauve represent the p-value of the enrichment. **Supplementary Table S1.** Summary of RNA-Seq data quality analysis of *H. discus hannai* (DD) and hybrid *H. discus hannai* ♀ × *H. fulgens* ♂ (DF) during short-term low salinity exposure 

## Data Availability

The RNA sequencing data used in this study has been uploaded to the National Center for Biotechnology Information (NCBI) as BioProject ID: PRJNA799755. The SRA accessions are SRR17720355-SRR17720372. The available link for the BioProject’s metadata is https://dataview.ncbi.nlm.nih.gov/object/PRJNA799755?reviewer=hpjsh10gb026f1snfuusm00ugk
