# Supplementary material for: Transcriptome analysis reveals fluid shear stress (FSS) and atherosclerosis pathway as a candidate molecular mechanism of short-term low salinity stress tolerance in abalone
Source: BMC Genomics. 2022 May 23;23:392. doi: 10.1186/s12864-022-08611-8 (PMC9128277; doi:10.1186/s12864-022-08611-8)
Supplement: Supplementary file 1 — Additional file 1: Supplementary Figure 1. Venn diagram of common and differentially expressed mRNAs of gill tissues of abalone during short-term low salinity exposure: H. discus hannai (DD) and hybrid H. discus hannai ♀ × H. fulgens ♂ (DF). A) Comparisons within DD (CDvsD3, CDvsD24, D3vsD24), B) Comparisons within DF (CFvsF3, CFvsF24, F3vsF24), and C) Comparisons between DD and DF (CDvsCF, D3vsF3, D24vsF24). Controls: CD, CF; 3 h at low salinity exposure: D3, F3; and 24 h at low salinity exposure: D24, F24. Supplementary Fig. 2. Volcano plots of the differentially expressed genes (DEGs) of gill tissues of abalone during short-term low salinity exposure: I) H. discus hannai (DD): A. Comparison in DD between control (CD) and low salinity after 3 h (D3), B. Comparison in DD between control (CD) and low salinity after 24 h (D24). II) Hybrid H. discus hannai ♀ × H. fulgens ♂ (DF). A. Comparison in DF between control (CF) and low salinity after 3 h (F3) B. Comparison in DF between control (CF) and low salinity after 24 h (D24). Red color denotes up-regulated genes and Blue color denotes and down-regulated. Supplementary Fig. 3. Comparison of top 20 Kyoto Encyclopedia of Gene and Genome (KEGG) pathways enrichment statistics of gill tissues of abalone during short-term low salinity exposure. H. discus hannai (DD) and hybrid H.discus hannai ♀ × H. fulgens ♂ (DF). FSS pathway is highlighted with a rectangular red box. (I) Enrichment analysis for DD: A. Comparison between control (CD) and low salinity after 3 h (D3), B. Comparison between control (CD) and low salinity after 24 h (D24), and C. Comparison between low salinity groups after 3 h and 24 h (CD3vsD24). (II) Enrichment analysis for DF: A. Comparison between control (CF) and low salinity group after 3 h (F3), B. Comparison between control (CF) and low salinity group after 24 h (F24), and C. Comparison between low salinity groups after 3 h and 24 h (F3vsF24). The size of each point represents the number of genes annotat [file 12864_2022_8611_MOESM1_ESM.docx]

Figure Legend


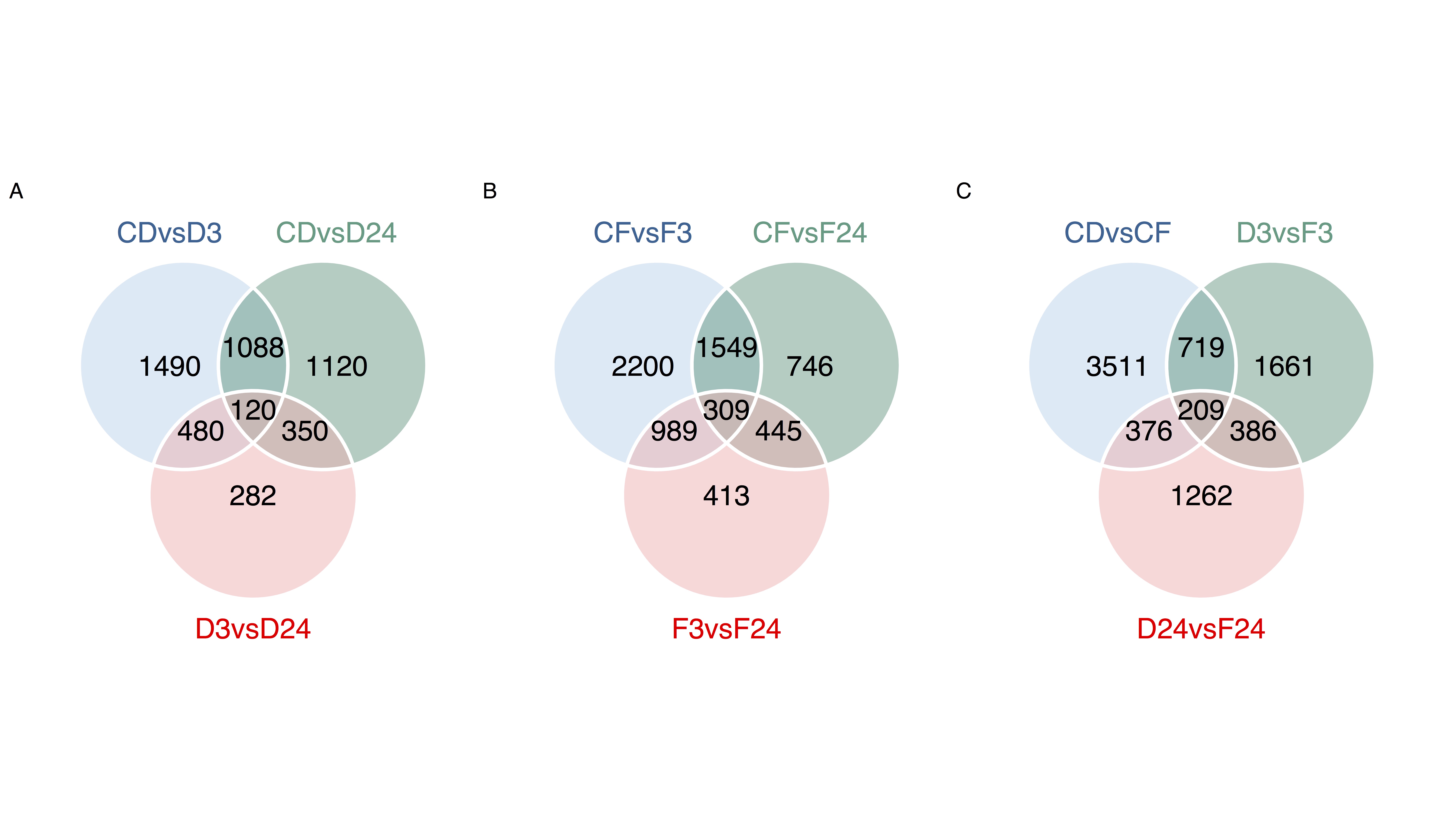


Supplementary Figure 1. Venn diagram of common and differentially expressed mRNAs of gill tissues of abalone during short-term low salinity exposure: *H. discus hannai* (DD) and hybrid *H. discus hannai* ♀ × *H. fulgens* ♂ (DF). **A)** Comparisons within DD (CDvsD3, CDvsD24, D3vsD24), **B)** Comparisons within DF (CFvsF3, CFvsF24, F3vsF24), and **C)** Comparisons between DD and DF (CDvsCF, D3vsF3, D24vsF24). Controls: CD, CF; 3 h at low salinity exposure: D3, F3; and 24 h at low salinity exposure: D24, F24.

**I.**


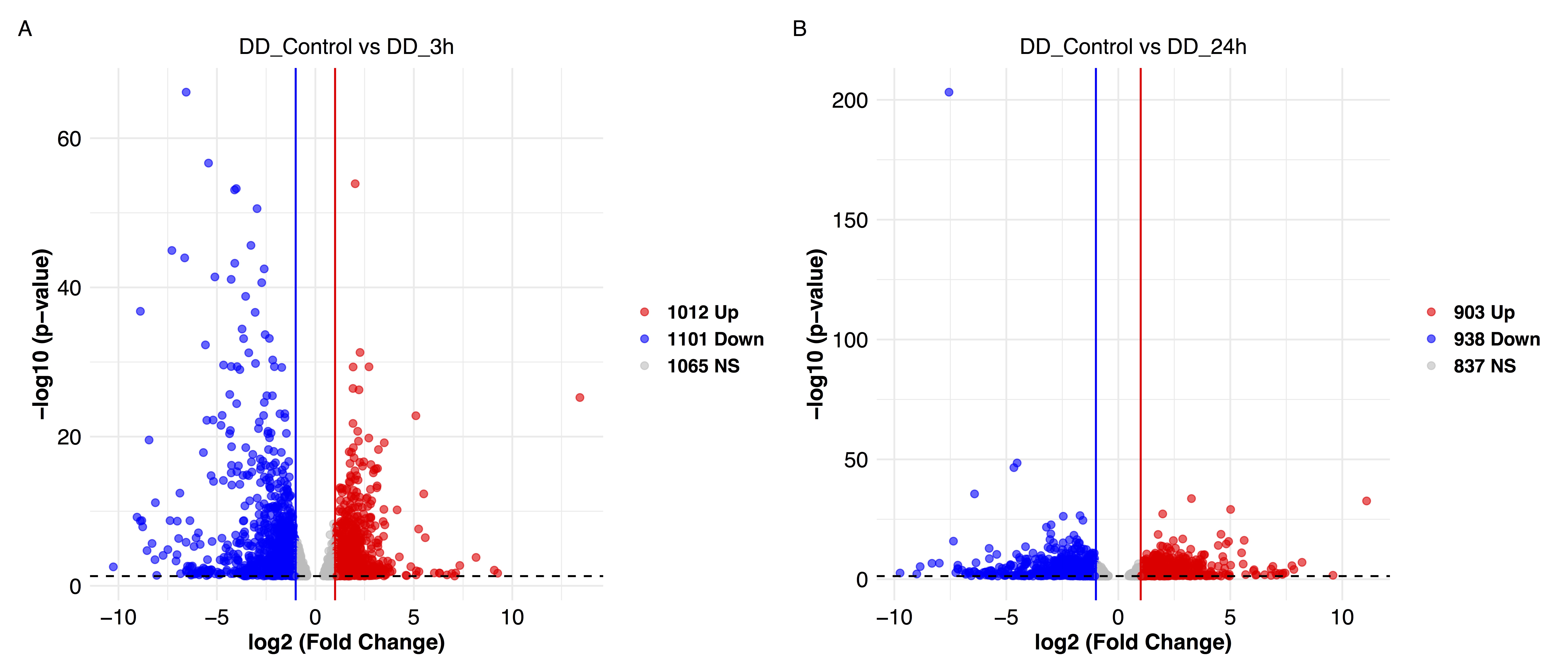


II.


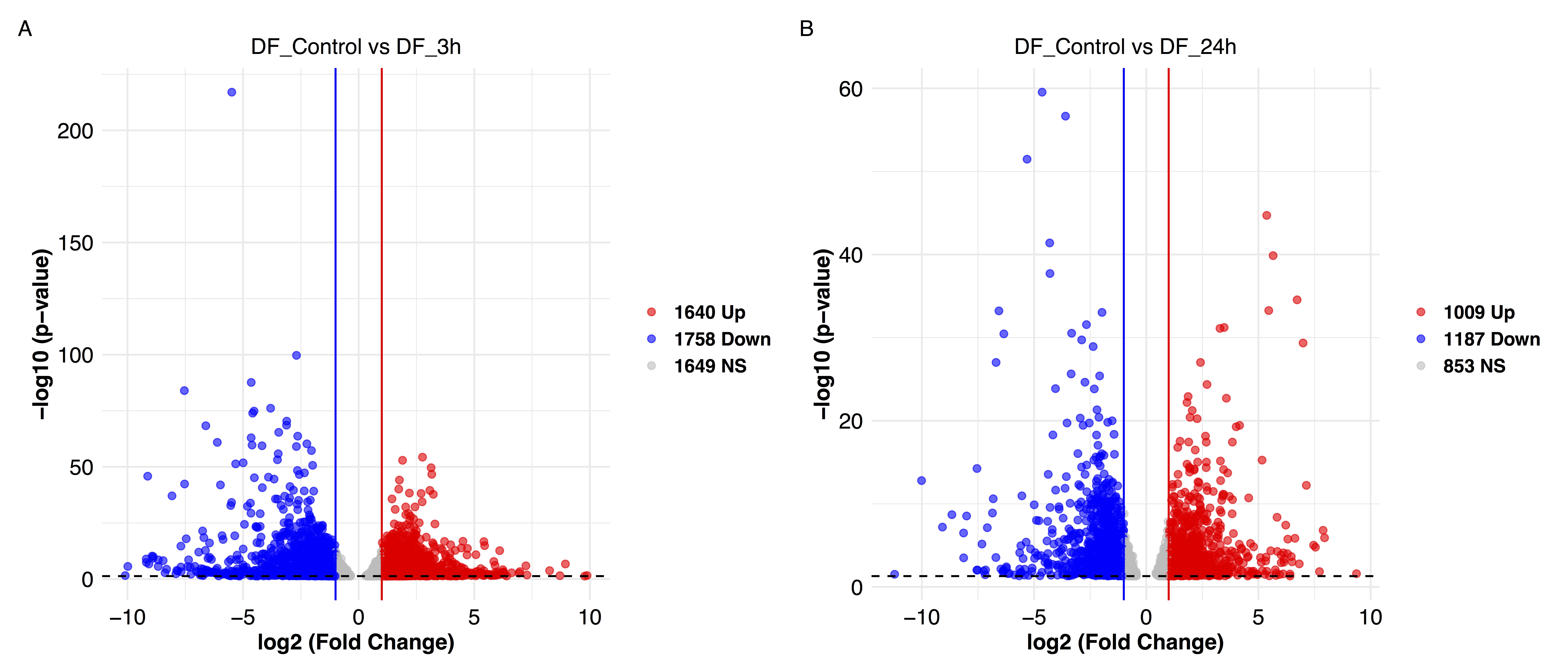


Supplementary Fig. 2. Volcano plots of the differentially expressed genes (DEGs) of gill tissues of abalone during short-term low salinity exposure: **I**) *H. discus hannai* (DD): **A.** Comparison in DD between control (CD) and low salinity after 3 h (D3), **B**. Comparison in DD between control (CD) and low salinity after 24 h (D24).

**II**) Hybrid *H. discus hannai* ♀ × *H. fulgens* ♂ (DF). **A**. Comparison in DF between control (CF) and low salinity after 3 h (F3) **B**. Comparison in DF between control (CF) and low salinity after 24 h (D24). Red color denotes up-regulated genes and Blue color denotes and down-regulated.

**I.**


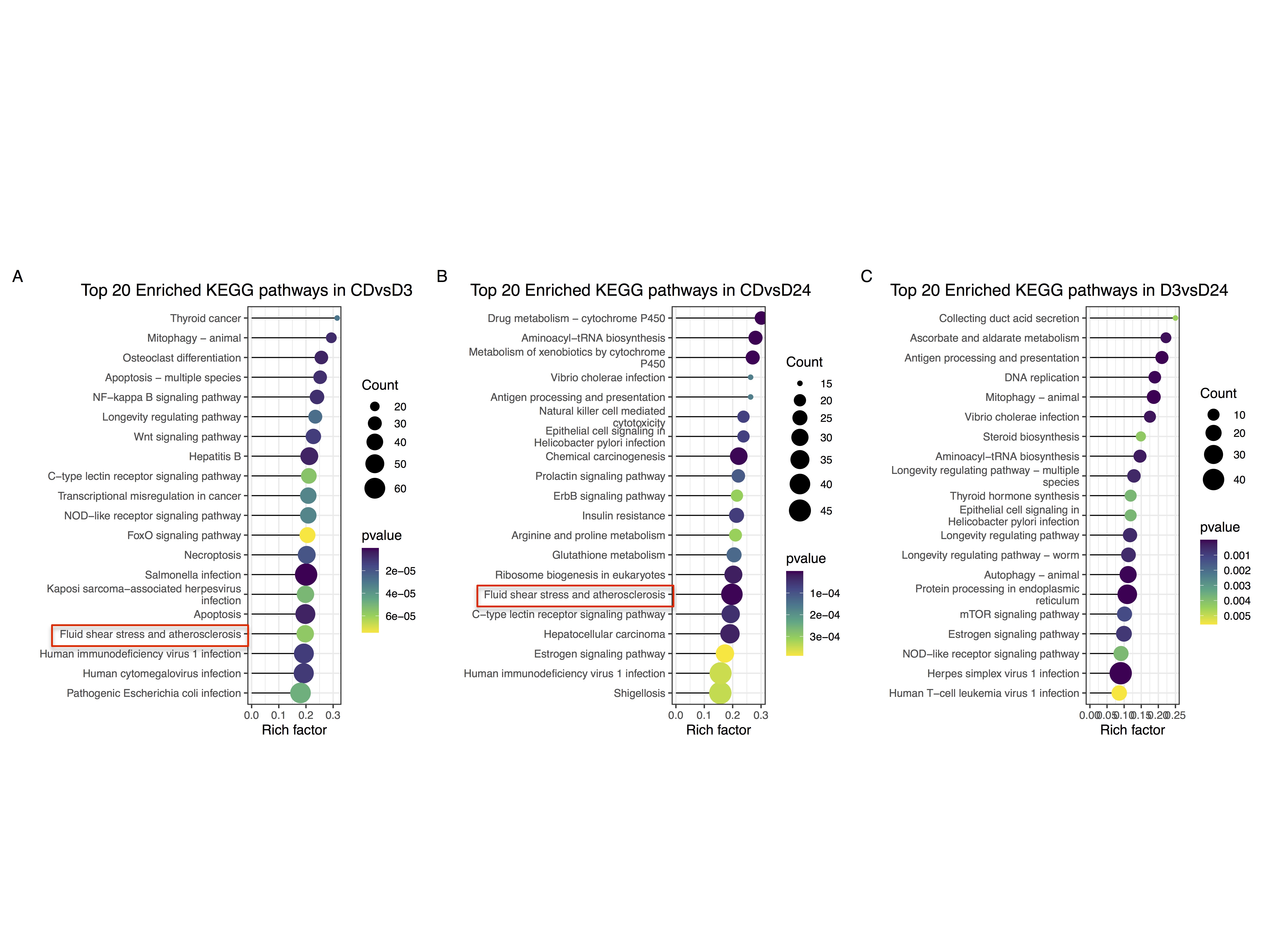


**II.**


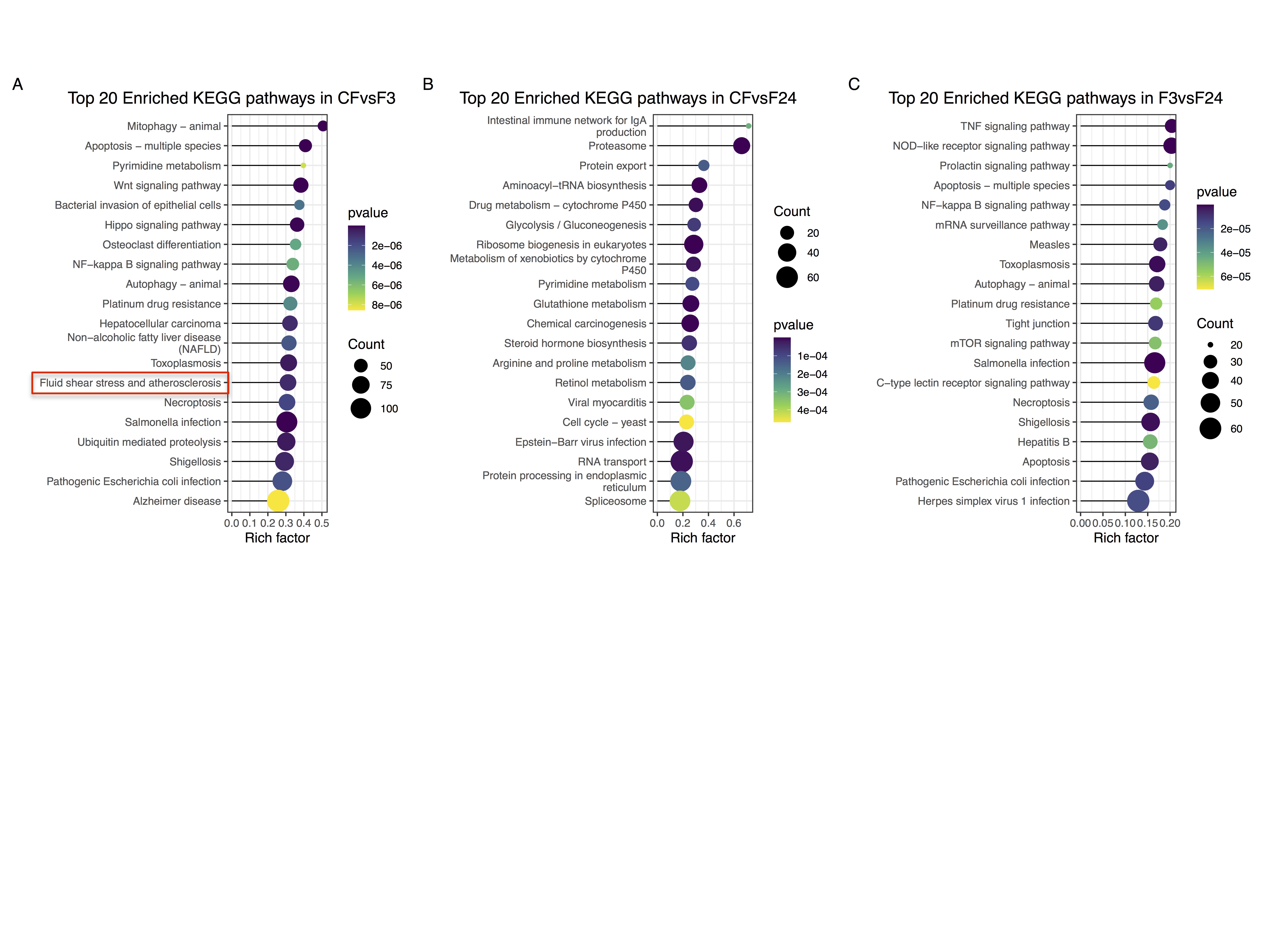


Supplementary Fig. 3. Comparison of top 20 Kyoto Encyclopedia of Gene and Genome (KEGG) pathways enrichment statistics of gill tissues of abalone during short-term low salinity exposure. *H. discus hannai* (DD) and hybrid *H. discus hannai* ♀ × *H. fulgens* ♂ (DF). FSS pathway is highlighted with a rectangular red box. **(I)** Enrichment analysis for DD: **A**. Comparison between control (CD) and low salinity after 3 h (D3), **B.** Comparison between control (CD) and low salinity after 24 h (D24), and **C.** Comparison between low salinity groups after 3 h and 24 h (CD3vsD24).

**(II)** Enrichment analysis for DF: **A**. Comparison between control (CF) and low salinity group after 3 h (F3), **B.** Comparison between control (CF) and low salinity group after 24 h (F24), and **C.** Comparison between low salinity groups after 3 h and 24 h (F3vsF24). The size of each point represents the number of genes annotated to the KEGG pathway. Different colors from yellow to mauve represent the p-value of the enrichment.

List of Tables

Supplementary Table S1: Summary of RNA-Seq data quality analysis of *H. discus hannai* (DD) and hybrid *H. discus hannai* ♀ × *H. fulgens* ♂ (DF) during short-term low salinity exposure

| Species | Sample | Raw reads | Clean reads | Clean bases (Gp) | Q20 (%) | Q30 (%) | Total mapped reads | Unique mapped reads |
| --- | --- | --- | --- | --- | --- | --- | --- | --- |
| DD | CDD1 | 44,927,760 | 42,979,310 | 6.45 | 97.97 | 94.22 | 36,581,148 (85.11%) | 33,926,097 (78.94%) |
|  | CDD2 | 45,551,022 | 42,782,156 | 6.42 | 98.14 | 94.62 | 36,046,478 (84.26%) | 33,399,443 (78.07%) |
|  | CDD3 | 46,584,560 | 44,026,448 | 6.60 | 98.01 | 94.29 | 37,585,664 (85.37%) | 34,920,606 (79.32%) |
|  | DD3h1 | 45,560,598 | 43,353,010 | 6.50 | 97.94 | 94.18 | 36,736,671 (84.74%) | 34,005,683 (78.44%) |
|  | DD3h2 | 45,400,358 | 43,620,946 | 6.54 | 98.09 | 94.45 | 37,226,253 (85.34%) | 34,576,741 (79.27%) |
|  | DD3h3 | 46,589,958 | 44,566,126 | 6.68 | 97.66 | 93.52 | 38,146,536 (85.6%) | 351,769,66 (78.93%) |
|  | DD24h1 | 46,724,866 | 45,074,510 | 6.76 | 97.25 | 92.58 | 38,285,827 (84.94%) | 35,681,240 (79.16%) |
|  | DD24h2 | 47,918,846 | 45,632,730 | 6.84 | 97.98 | 94.31 | 38,936,129 (85.33%) | 36,036,780 (78.97%) |
|  | DD24h3 | 46,510,206 | 44,415,264 | 6.66 | 97.91 | 94.10 | 37,524,717 (84.49%) | 34,833,922 (78.43%) |
| DF | CDF1 | 46,968,304 | 44,590,554 | 6.69 | 97.85 | 93.64 | 28,350,051 (63.58%) | 26,345,466 (59.08%) |
|  | CDF3 | 46,248,978 | 44,203,864 | 6.63 | 97.31 | 92.73 | 27,858,871 (63.02%) | 25,984,810 (58.78%) |
|  | CDF4 | 45,684,772 | 43,747,726 | 6.56 | 97.77 | 93.31 | 28,442,789 (65.02%) | 26,437,283 (60.43%) |
|  | DF3h1 | 47,965,708 | 46,387,736 | 6.96 | 97.75 | 93.36 | 29,656,054 (63.93%) | 27,643,123 (59.59%) |
|  | DF3h2 | 46,723,942 | 45,013,444 | 6.75 | 97.98 | 94.24 | 29,342,837 (65.19%) | 27,318,282 (60.69%) |
|  | DF3h3 | 45,227,282 | 43,638,762 | 6.55 | 97.96 | 94.21 | 28,240,234 (64.71%) | 26,266,388 (60.19%) |
|  | DF24h1 | 45,417,254 | 43,576,566 | 6.54 | 97.87 | 93.89 | 27,772,621 (63.73%) | 25,942,243 (59.53%) |
|  | DF24h2 | 43,093,600 | 41,328,914 | 6.20 | 98.09 | 94.49 | 26,817,412 (64.89%) | 25,001,900 (60.49%) |
|  | DF24h3 | 43,5713,80 | 42,076,870 | 6.31 | 97.93 | 94.11 | 27,389,918 (65.09%) | 25,625,672 (60.9%) |
